# Network Pharmacology and Molecular Docking-Based Mechanism Study to Reveal Antihypertensive Effect of Gedan Jiangya Decoction

**DOI:** 10.1155/2022/3353464

**Published:** 2022-08-22

**Authors:** Hanxing Liu, Shadi A. D. Mohammed, Fang Lu, Pingping Chen, Yu Wang, Shumin Liu

**Affiliations:** ^1^Graduate School of Heilongjiang University of Chinese Medicine, Harbin, 150040 Heilongjiang, China; ^2^Institute of Traditional Chinese Medicine, Heilongjiang University of Chinese Medicine, Harbin, 150040 Heilongjiang, China

## Abstract

Primary hypertension is understood as a disease with diverse etiology, a complicated pathological mechanism, and progressive changes. Gedan Jiangya Decoction (GJD), with the patent publication number CN114246896A, was designed to treat primary hypertension. It contains six botanical drugs; however, the underlying mechanism is uncertain. We utilized network pharmacology to predict the active components, targets, and signaling pathways of GJD in the treatment of primary hypertension. We also investigated the potential molecular mechanism using molecular docking and animal experiments. The Traditional Chinese Medicine System Pharmacology Database and Analysis Platform (TCMSP), the Protein Database (UniProt), and a literature review were used to identify the active components and related targets of GJD's pharmacological effects. The GeneCards, Online Mendelian Inheritance in Man (OMIM), Therapeutic Target Database (TTD), and DrugBank databases were utilized to identify hypertension-related targets. Based on a Venn diagram of designed intersection targets, 214 intersection targets were obtained and 35 key targets for the treatment of hypertension were determined using the STRING data platform and Cytoscape software. The Kyoto Encyclopedia of Genes and Genomes (KEGG) enrichment analysis of key targets revealed that the relevant molecular action pathways of GJD in the treatment of hypertension include the Toll-like receptor, MAPK, PI3K-Akt, and renin-angiotensin signaling pathways. A GJD active ingredient-key target-pathway connection diagram was created using Cytoscape software, and 11 essential active components were selected. Molecular docking was then used to verify the binding activity of key targets and key active ingredients in GJD to treat primary hypertension. The results of this study indicate that AGTR1, AKT1 with puerarin, EDNRA with tanshinone IIA, MAPK14 with daidzein, MAPK8 with ursolic acid, and CHRM2 with cryptotanshinone had high binding activity to the targets with active components, whereas AGTR1 was selected as target genes verified by our experiment. HPLC was utilized to identify the five active ingredients. Experiments in high-salt rats demonstrated that GJD might decrease the expression of AGTR1 in the kidney and thoracic aorta while increasing the expression of eNOS by preventing the activation of the renin-angiotensin pathway, thereby reducing lowering systolic and diastolic blood pressure.

## 1. Introduction

Hypertension is well-defined as a high systemic arterial blood pressure induced by the environment, polygenic heredity, and several risk factors. It has a great influence on the structure and function of blood vessels and often leads to brain, renal, and heart complications. It is also a major risk factor for cardiovascular diseases such as coronary heart disease, left ventricular hypertrophy, and arrhythmia [1]. Hypertension is classified into two major groups, which are primary and secondary hypertension, with primary hypertension responsible for 90% of hypertension patients [2]. The occurrence of hypertension in China increases yearly due to the changes in the environment and their social lifestyle. A recent study [3] revealed that 44.7% of Chinese people within the age range of 35 to 75 are reported to have high blood pressure, with over 260 million hypertensive people having uncontrolled blood pressure and less than one-third getting treatment. As such, the treatment and prevention of high blood pressure are important priorities.

Traditional Chinese medicine (TCM) is a combination of a complex herbal formula that has been used for thousands of years to treat countless diseases and increase life expectancy in various parts of the world. The complex interaction of several bioactive components in herbal preparations or medicine results in the synergistic therapeutic effect of TCM [4]. When treating hypertension, TCM has the advantages of stability, multiefficacy, prospective, multitarget, improved cardiovascular remodeling, synergistic blood pressure reduction, improving vascular endothelial function, reducing toxicity, protecting target organs, and increasing efficiency [5–7]. TCM has been mostly used at the grassroots level in China to treat and prevent hypertension. It stresses the concept of therapy and differentiation from a general perspective, which is highly satisfactory in controlling various risk factors of hypertension and decreasing the overall cardiovascular risk [8]. However, establishing its efficiency through scientific experiments and analyzing its molecular mechanism remain a great challenge.

In recent years, network pharmacology, based on bioinformatics and systems biology, has been widely used in TCM research to clarify the TCM treatment of complex ailments from a multicomponent, multitarget, multipathway approach [9]. The primary molecular mechanism of network pharmacology is expected to become an essential direction in TCM research. This method represents the interaction of drug components, targets, and diseases in a network form, revealing the molecular mechanism of different active ingredients and providing new methods and ideas for understanding the processes or pathways of disease medication treatment [10]. The molecular docking technique simulates ligand-receptor docking to predict the affinity and binding mechanism between therapeutic and target molecules. Molecular docking is utilized to understand the relation between ligands and receptors and the manufacturing and design of new medications [11, 12]. Currently, the joint use of network pharmacology and molecular docking has been efficiently applied to the study of TCM and its compound prescriptions [13–15].

Gedan Jiangya Decoction (GJD; patent publication number: CN114246896A) includes six botanical drugs: Uncaria rhynchophylla (Miq.) Miq. ex Havil. (Gouteng), Salvia miltiorrhiza Bunge (Danshen), Pueraria lobata (Willd.) Ohwi. (Gegen), Eucommia ulmoides Oliv. (Duzhong), Prunella vulgaris L. (Xiakucao), and Achyranthes bidentata Blume (Niuxi). Previous studies have revealed that isorhynchophylline and rhynchophylline in Uncaria rhynchophylla can reflexively and directly inhibit the vasomotor center, dilating peripheral blood vessels as exertion antihypertensive effects by inhibiting the influx of extracellular Ca^2+^ [16, 17]. Studies have also revealed that both Salvia miltiorrhiza and Pueraria lobata have a wide variety of pharmacological effects on the cardiovascular system, including anti-inflammatory, endothelial cell function protection, antioxidant, vasodilation, and myocardial protection [18–21]. Prunella vulgaris, Achyranthes bidentata, and Eucommia ulmoides also have therapeutic benefits for cardiovascular diseases such as hypertension [22–24].

Previous studies have mostly concentrated on a single medication or ingredient, making it challenging to precisely represent the mechanism of GJD's multichannel, multitarget, and multicomponent compound. Therefore, this research seeks to use the study methods of molecular docking and network pharmacology to produce the GJD active ingredient-key target-pathway, analyze the molecular mechanism of GJD in treating hypertension, and provide a reference for future medical clinical experiments and theoretical research.

## 2. Materials and Method

### 2.1. Network Pharmacology Analysis

#### 2.1.1. GJD Active Ingredients, Screening, and Target Prediction

The TCMSP database (https://old.tcmsp-e.com/index.php) [25] was used to collect the active components of Uncaria rhynchophylla, Salvia miltiorrhiza, Pueraria lobata, Eucommia ulmoides, Prunella vulgaris, and Achyranthes bidentata, and the active components were preliminarily evaluated based on the two ADME properties of oral bioavailability (OB) ≥ 30% and drug-likeness (DL) ≥ 0.18. Simultaneously, the screened components were complemented using published literature to obtain the GJD active ingredients. The TCMSP database is used to predict the target of active ingredients. The UniProt database (https://www.uniprot.org/) [26] is then utilized to standardize protein target information and screen the targets with “Reviewed” and “Human” as screening conditions and complete the transformation from protein name to gene name to obtain the relevant target of GJD.

#### 2.1.2. Prediction of Primary Hypertension Targets

We used the GeneCards database (https://www.genecards.org/), the OMIM database (https://omim.org/), the TTD database (http://db.idrblab.net/ttd/) [27], and the DrugBank database (https://go.drugbank.com/) [28] to collect potential primary hypertension targets with “primary hypertension” as the keyword. The higher the score value in the GeneCards database, the more closely connected the target is to the disease. To obtain the potential primary hypertension target, first, we select the target with a score value more than the median and then select the remaining target with a higher score than the median. Then, combine the primary hypertension-related targets from the four disease databases mentioned above and remove duplicates to get the primary hypertension-related targets.

#### 2.1.3. Protein-Protein Interaction Network

The GJD-related and primary hypertension-related targets were imported into the R language, and the intersection of the targets was drawn as a Venn diagram. Then, import the intersection targets into the STRING 11.0 data platform [29], setting the biological species to “Homo sapiens” and the minimum interaction score to “highest confidence” (>0.9), and hide the free isolates node to build a protein-protein interaction (PPI) network model. After that, use the Cytoscape 3.8.0 [30] tool to calculate and screen the degree value, and choose the intersection target with a degree value greater than twice the median as the key target for primary hypertension treatment.

#### 2.1.4. GO and KEGG Pathway Enrichment Analyses

We import key targets into the Metascape data platform (https://metascape.org) [31], select “H. sapiens” for “Input as species” and “Analysis as species” conditions, and then select “custom analysis” with *P* < 0.01 as the screening condition; GO function analysis and KEGG pathway enrichment analysis are performed for key targets; and the enrichment results are drawn into a bubble plot in the R language.

#### 2.1.5. Network Construction and Analysis of Target Pathway

The active components corresponding to the key targets were screened out, and the drugs, key targets, and KEGG pathways were transferred into Cytoscape program for analysis, and the network diagram of the GJD active ingredient-key target-pathway was obtained. Use the “Analyze Network” of the Cytoscape toolbar to calculate it, and select the active ingredients with a degree value greater than 2 times the median as the key ingredients in treating primary hypertension.

### 2.2. Molecular Docking (Key Ingredients to Key Targets)

Using the PDB database (http://www.rcsb.org/) [32] to download the three-dimensional protein structure of key targets, select the species source as “Homo sapiens,” and the resolution is high (resolution < 3.0 Å) and the protein with the crystal structure of the original complex ligand, and save it as a PDB format file; use Discovery Studio to preprocess it, including deleting water molecules, hydrogenation, charge distribution, and other operations to the original ligand, focusing on the spatial structure of the original ligand; create the active docking pocket using PyMOL's GetBox plugin; record the spatial location and radius of the active pocket; and then import it into AutoDockTools [33] and export it in PDBQT format. Then, get the core component SDF format file and convert it via the PubChem database (https://pubchem.ncbi.nlm.nih.gov/) [34]; save it in PDB format, input it into AutoDockTools, and output it as PDBQT format. Finally, AutoDock Vina [35] was used for molecular docking, while PyMOL and Discovery Studio were used for mapping.

### 2.3. Experiment

#### 2.3.1. Preparation of GJD

GJD's composition is as follows: Uncaria rhynchophylla (Miq.) Miq. ex Havil. (Gouteng), 10 g; Salvia miltiorrhiza Bunge (Danshen), 25 g; Pueraria lobata (Willd.) Ohwi (Gegen), 30 g; Eucommia ulmoides Oliv. (Duzhong), 15 g; Prunella vulgaris L. (Xiakucao), 15 g; and Achyranthes bidentata Blume (Niuxi), 20 g. The botanical drugs described above were purchased from Heilongjiang Xiushengtang Pharmaceutical Co., Ltd. First, mix the botanical drugs (Salvia miltiorrhiza, Pueraria lobata, Eucommia ulmoides, Prunella vulgaris, and Achyranthes bidentata) with 60% aqueous ethanol solution in a material liquid ratio of 1 g : 10 ml, soak for 0.5 hour, heat and reflux the soaking solution for 1.5 hours (keep the solution slightly boiling during the reflux process), and filter with six layers of degreasing gauze; after that, in the same way, reheat and reflux with 10 times the amount of 60% aqueous ethanol solution for 1.5 hours and filter; then, to get extracts of Salvia miltiorrhiza, Pueraria lobata, Eucommia ulmoides, Prunella vulgaris, and Achyranthes bidentata, combine two filtrates, distill ethanol under reduced pressure using a rotary evaporator, and then dry under reduced pressure and vacuum. Second, soak Uncaria rhynchophylla in a 70% aqueous ethanol solution in a 1 g : 10 ml ratio for 0.5 hour, then heat it to 65~75°C, and soak it for 2 hours. Filter it through six layers of degreasing gauze, then soak it in 10 times amount of 70% aqueous ethanol solution for 2 hours, filter it, then use the above method to get the extract of Uncaria rhynchophylla. Finally, combine the two extracts to make GJD.

#### 2.3.2. HPLC Analysis of GJD

This analysis was performed using the Waters Alliance e2695, Waters 2998 PDA Detector, and Diamonsil® C18 column (250 × 4.6 mm, 5 *μ*m). Puerarin (Lot No. 110752-201816, Purity: 95.4%), tanshinone IIA (Lot No. 110766-202022, Purity: 98.9%), daidzein (Lot No. 111502-202003, 99.3%), ursolic acid (Lot No. 110742-201823, Purity: 99.9%), and cryptotanshinone (Lot no. 110852-201807, Purity: 99.0%) were purchased from National Institutes for Food and Drug Control. Take a dosage of puerarin, tanshinone IIA, daidzein, ursolic acid, and cryptotanshinone, and dissolve it in methanol. Standard solutions with concentrations of 0.3, 0.25, 0.5, 0.5, and 0.3 mg/ml were prepared. Before HPLC analysis, 1 g of GJD extract powder is mixed with 10 ml of methanol and filtered through a 0.22 *μ*m filter. The mobile phase was acetonitrile (A) -0.1% aqueous phosphoric acid solution (B). The following is the gradient elution program: 0-14 min, 30-37% A; 14-17 min, 37-55% A; 17-29 min, 55-67% A; 29-40 min, 67-89% A; 40-48 min, 89-97% A; and, 48-54 min, 97-100% A; column temperature: 30°C; flow rate: 1.0 ml/min. The wavelength of detection was 210 nm. The injection volume was 10 *μ*l.

#### 2.3.3. Animals

Twenty-one male SD (Sprague Dawley) rats (body weight 170 ± 10 g) were acquired from Liaoning Changsheng Biotechnology Co., Ltd. (license number SYXK (Hei) 2018-007) and maintained at a constant temperature (22 ± 2°C), humidity (55 ± 5%), and lighting (12 : 12 hr light-dark cycle). One week before the experiment, rats were given unrestricted access to food and water to acclimatize to their surroundings. All experimental protocols were approved by the Animal Ethics Committee of Heilongjiang University of Chinese Medicine, with approval number 2020031203.

#### 2.3.4. Construction of High-Salt Diet-Induced Hypertension Model

The SD rats were randomly divided into 3 groups, 7 rats in the control group, 7 rats in the model group, and 7 rats in the GJD group. The rats in the control group were fed with standard chow. In contrast, the rats in the other groups were fed with 8% NaCl high-salt feed (7.5%NaCl + 92.5%common feed, purchased from Guangdong Provincial Medical Laboratory Animal Center, license number Guangdong Feed Certificate (2019) 05073) for 8 weeks with free access to water. The body weight of the rats in each group was recorded every two weeks. The systolic blood pressure (SBP) and diastolic blood pressure (DBP), as well as heart rate (HR), were monitored every two weeks by the noninvasive blood pressure measurement system ALC-NIBP (Shanghai Alcott Biotechnology Co., Ltd.) using the tail-cuff method. Each rat was measured three times, and the average value was recorded. A long-term 8% high-salt diet for 8 weeks caused hypertension in rats [36]; rats with SBP of ≥160 mmHg were considered to have successfully established a hypertension model. All rats in the model and GJD groups were modeled successfully.

#### 2.3.5. Drug Treatment and Blood Pressure Measurement

For four weeks, each rat in the GJD group received 1.5 ml of GJD extract 1.07 g/kg/d by gavage, whereas the other groups received the same amount of 0.9% normal saline by gavage. The body weight of the rats in each group was recorded every two weeks. Rats' SBP, DBP, and HR were recorded every two weeks using the noninvasive blood pressure measurement system (ALC-NIBP). Each rat was measured three times, and the average value was recorded.

#### 2.3.6. Sample Preparation

All rats were anesthetized with an intraperitoneal injection of 3% sodium pentobarbital (45 mg/kg), and blood samples were obtained from the abdominal aorta and put in disposable blood collection tubes. Centrifuge at 3500 rpm for 15 minutes at 4°C, then extract serum and put it in a centrifuge tube. Hearts were removed and washed in cold saline. The heart's left ventricle is obtained by resecting the atrial free wall, major arteries, and right ventricle along the atrioventricular junction. Left ventricular mass index (LVMI) is calculated by the following formula: LVMI = left ventricular weight (mg)/body weight (g). At the same time, thoracic aorta and kidney tissues were taken and frozen at -80°C for detection using RT-qPCR, Western blot, and kits.

#### 2.3.7. ELISA Kit Index Determination

The ELISA Kit was used to measure the expression levels of renin, Ang II, ALD, and NO in serum and Ang II in renal tissue (Ang II kit was purchased from Jiangsu Jingmei Biotechnology Co., Ltd., and ALD, renin, and NO (nitrate reductase method) kit was purchased from Nanjing Jiancheng Bioengineering Institute). The procedure was carried out in strict accordance with the kit's instructions.

#### 2.3.8. RT-qPCR

The total RNA was extracted from kidney and thoracic aorta tissues using Trizol reagent (Takara), reverse transcribed into cDNA using PrimeScript RT Kit (Takara), and then subjected to real-time PCR reaction using TB Green® Premix Ex Taq™ II (Tli RNaseH Plus) (Takara), and the gene expression level was quantified using QuantStudio™3 Real-Time PCR system. The 2^-*ΔΔ*CT^ method was used to calculate and analyze the data, with GAPDH acting as an internal control. The following primers were used for PCR amplification Table 1.

#### 2.3.9. Western Blot Analysis

The total protein was obtained from the kidney and thoracic aorta using RIPA lysis buffer with PMSF and phosphatase inhibitor (Servicebio, China), and protein concentration was measured using a BCA protein detection kit (Servicebio, China). SDS-PAGE was used to separate the whole protein, which was subsequently transferred to 0.45 *μ*m on the PVDF membrane (Servicebio, China). The membrane was sealed with 5% nonfat milk at room temperature for 1 hour before incubating with primary antibody overnight at 4°C. The membrane was washed three times with TBST and incubated with a secondary antibody at room temperature for 1 hour, and the protein was identified using an ECL reagent (Servicebio, China). Primary antibodies include AGTR1 (1 : 1,000, Boster, China), eNOS (1 : 1,000, Servicebio, China), and *β*-actin (1 : 2,000, Servicebio, China).

#### 2.3.10. Statistical Analysis

All data in this experiment were statistically analyzed using the SPSS 26.0 software; measurement data were represented as mean ± standard deviation (mean ± SD), and one-way analysis of variance (ANOVA) was used to compare multiple groups. For variance homogeneity of pairwise comparisons between groups, the LSD test was employed, and Tamhane's T2 (M) test was used for variance heterogeneity. *P* < 0.05 was regarded as statistically significant. The statistical analysis findings were created using the GraphPad Prism 7.0 program.

## 3. Results

### 3.1. Network Pharmacology Analysis

#### 3.1.1. GJD Target Prediction Results in Primary Hypertension Treatment

**Figure 1:**
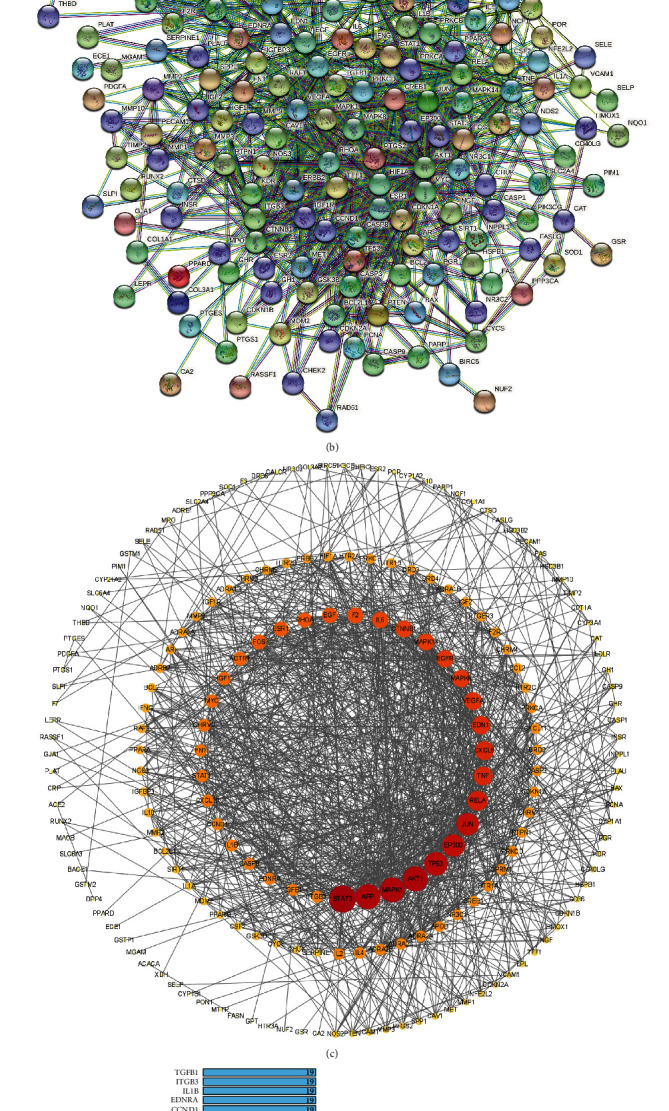
(a) According to a Venn diagram, GJD and primary hypertension have 214 common targets that might be utilized as therapeutic targets. (b) Target protein interaction network (PPI). (c) Target protein interaction network analysis in Cytoscape software. (d) The top 35 potential targets of PPI. As the degree value increases from small to large, the node's color changes from yellow to red, and the node's size changes from small to large.

We obtained 32 active ingredients of Uncaria rhynchophylla, 59 active ingredients of Salvia miltiorrhiza, 4 active ingredients of Pueraria lobata, 26 active ingredients of Eucommia ulmoides, 11 active ingredients of Prunella vulgaris, and 17 active ingredients of Achyranthes bidentata through ADME screening and the TCMSP database, and according to the literature review, there is one active ingredient of Salvia miltiorrhiza [37, 38], two active ingredients of Pueraria lobata [39, 40], and two active ingredients of Prunella vulgaris [23, 41]. Finally, 154 GJD active ingredients were obtained, and 322 predicted targets for GJD active ingredients were collected using the TCMSP database. We obtained 2236 hypertension-related targets from the GeneCards database, 126 from the OMIM database, 107 from the TTD database, and 43 from the DrugBank database. The primary hypertension-related targets collected from the four databases mentioned above were merged together, and duplicate targets were removed, yielding 2340 potential primary hypertension targets. Using the R language, 322 GJD-related targets and 2340 primary hypertension-related targets were intersected, and a Venn diagram was drawn, giving 214 intersected targets, as shown in Figure 1(a).

#### 3.1.2. Constructing of PPI Network and Acquiring Key Targets

As shown in Figure 1(b), the GJD target protein-protein interaction (PPI) network for treating primary hypertension was created by importing the intersection target into the STRING data platform. Cytoscape was used to visualize it (see Figure 1(c)), and there are 199 nodes and 1145 edges in total. Thirty-five key targets with a degree value greater than 18 (2 times the median) were obtained, which may be the targets for GJD to play an essential role in primary hypertension treatment (see Figure 1(d)).

#### 3.1.3. GO and KEGG Analysis Results

The key targets were imported into the Metascape data platform, and the results of the GO enrichment function analysis showed that the targets were mainly involved in biological processes such as blood vessel development, vasculature development, cardiovascular system development, blood vessel morphogenesis, and angiogenesis; the main related molecular functions are transcription factor binding, cytokine receptor binding, receptor regulator activity, receptor-ligand activity, cytokine activity, and nitric-oxide synthase regulator activity.

**Figure 2:**
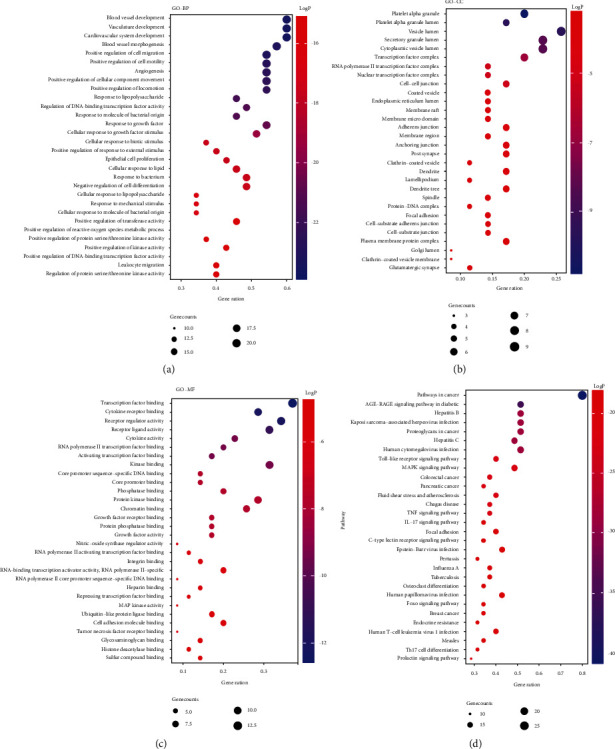
(a) The top 30 biological processes. (b) The top 30 cellular components. (c) The top 30 molecular functions. (d) The top 30 KEGG pathways.

A total of 149 pathways were obtained from the KEGG pathway enrichment analysis. Among them are the Toll-like receptor signaling pathway, PI3K-Akt signaling pathway, MAPK signaling pathway, TNF signaling pathway, NOD-like receptor signaling pathway, NF-*κ*B pathway, and renin-angiotensin pathway which are strongly correlated with hypertension. According to the enrichment analysis results, according to the Log*P* value from small to large, the top 30 items of GO-BP, GO-CC, GO-MF, and KEGG were selected, respectively, and visualized by using the R language, as shown in Figure 2.

#### 3.1.4. Network Construction of Target Pathway and Key Ingredient Acquisition

**Figure 3:**
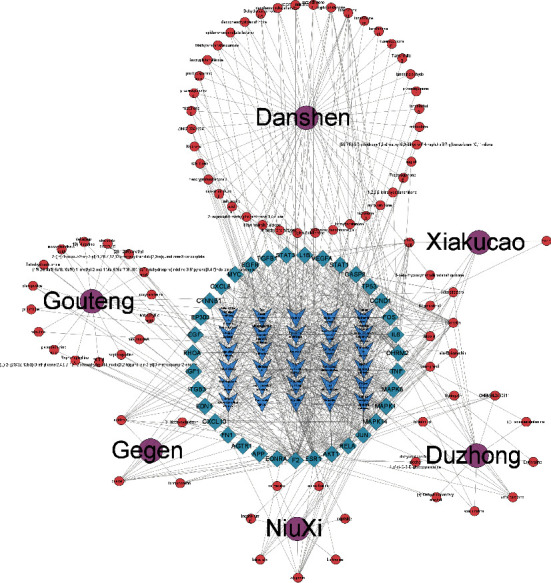
Using Cytoscape, construct the GJD active ingredient-key target-pathway network. 11 key ingredients were screened for potential treatment of primary hypertension. The purple circles in the outer circle represent the 6 Chinese herbal medicines in GJD, the red circles in the outer circle represent the active components of each Chinese herbal medicine, the blue diamonds in the inner circle represent the core targets, and the blue arrows in the inner circle represent the KEGG pathway.

The GJD active ingredient-key target-pathway network diagram was constructed using Cytoscape (see Figure 3), and through the “Analyze Network” tool of Cytoscape, 11 key ingredients of GJD in treating primary hypertension are screened, as shown in Supplementary Material (2).

### 3.2. Molecular Docking Result

**Figure 4:**
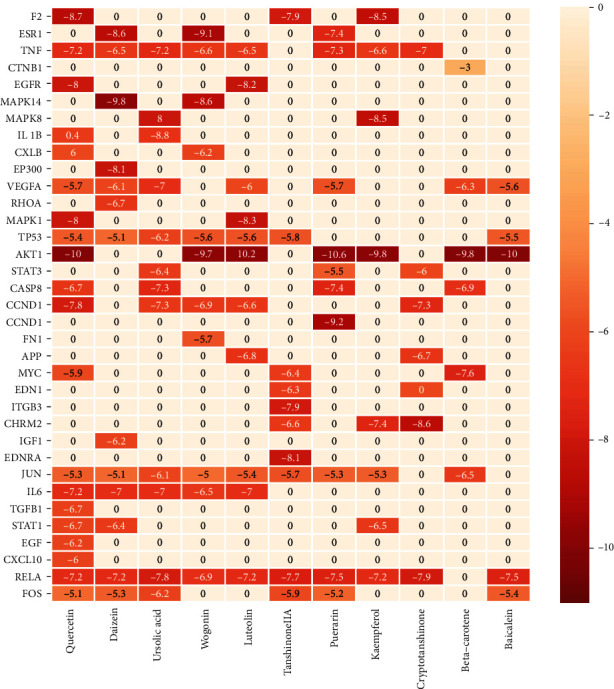
Heatmap of docking scores for 35 target proteins with 11 active ingredient molecules. The darker the color, the stronger the binding activity. The score of 0 means they are not molecularly docked.

**Figure 5:**
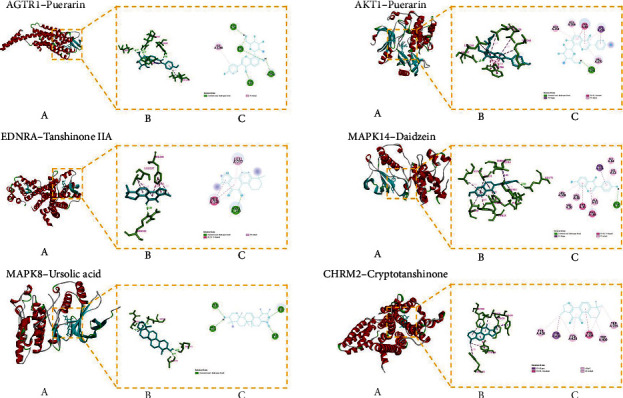
The molecular docking pattern. Each target protein with its active ingredient molecular docking pattern diagram is divided into three little figures (a), (b), and (c). (a) Represents the overall binding position of small molecule compounds (sticks model) on related proteins (cartoon model). (b) Shows the interaction of small molecule compounds (blue) with important residues (green) on related proteins. (c) Displays a two-dimensional plan, which is convenient for observing the hydrogen bonding and hydrophobic interactions between small molecule compounds and protein residues.

The target protein's and active ingredient molecules' docking results are shown in Figure 4, and these target proteins' PDB ID are shown in Table 2. The results showed that binding energies below -5.0 kcal·mol^−1^ represented 98% of the targets, indicating that most of the targets had strong binding activity to the active ingredients, demonstrating the reliability of the network pharmacology prediction results. Among them, AGTR1, AKT1 with puerarin, EDNRA with tanshinone IIA, MAPK14 with daidzein, MAPK8 with ursolic acid, and CHRM2 with cryptotanshinone all have a strong correlation with GJD in the treatment of primary hypertension.  Figure 5 illustrates their docking pattern. The molecular docking results indicated that the active ingredients of GJD, puerarin, have significant binding activity with the primary hypertension targets AGTR1. However, further experiments are needed to verify their strong interaction.

### 3.3. Result of HPLC Analysis

HPLC detected five key active ingredients of GJD, including puerarin, tanshinone IIA, daidzein, ursolic acid, and cryptotanshinone. Supplementary Material (1) shows the findings of HPLC analysis of these five active ingredients.

### 3.4. Experiment Verification

#### 3.4.1. Effect of GJD on Blood Pressure and Heart Rate (HR)

**Figure 6:**
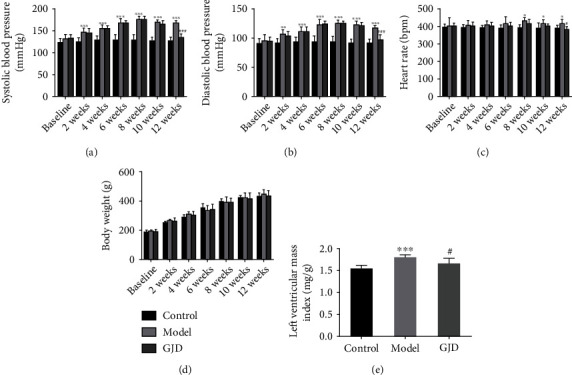
SBP, DBP, HR, and body weight indexes of SD rats (*n* = 7) before high-salt modeling, 8-week high-salt modeling, and 4 weeks of GJD treatment and LVMI after 4 weeks of GJD treatment. (a) Systolic blood pressure. (b) Diastolic blood pressure. (c) Heart rate. (d) Body weight. (e) Left ventricular mass index. ^∗^*P* < 0.05, ^∗∗^*P* < 0.01, and ^∗∗∗^*P* < 0.001 as compared to the control group; ^#^*P* < 0.05, ^##^*P* < 0.01, and ^###^*P* < 0.001 as compared to the model group.

Elevated blood pressure is the main clinical manifestation of hypertension, and elevated heart rate is a common feature of individuals with hypertension [42, 43]. Therefore, we evaluated the effect of GJD on primary hypertension by measuring SBP, DBP, and HR in rats. As shown in Figures 6(a)–6(d), SD rats were fed 8% high-salt diet for 8 weeks, the SBP was 176.68 mmHg, the DBP was 125.62 mmHg in the model group, and the SBP was 176.86 mmHg in the GJD group, and the DBP was 125.28 mmHg, indicating that the hypertension model established is successful. After 4 weeks of treatment, the SBP was 168.95 mmHg, the DBP was 118.08 mmHg in the model group, the SBP was 135.31 mmHg, and the DBP was 97.69 mmHg in the GJD group. The SBP and DBP were significantly reduced in the GJD group (*P* < 0.001), indicating that GJD has a significant antihypertensive effect. In addition, the HR of the rats in the GJD group was also lower than that in the model group. (*P* < 0.05). By calculating the left ventricular mass/body weight of the rats in each group, after 4 weeks of treatment, the LVMI in the model group was significantly higher than that in the control group (*P* < 0.001), Figure 6(e), while that in the GJD group was decreased compared with the model group (*P* < 0.05). We also observed no difference in the body weight of rats between the groups during the same period (Figure 6(d)).

#### 3.4.2. Effect of GJD on RAAS and Serum NO

**Figure 7:**
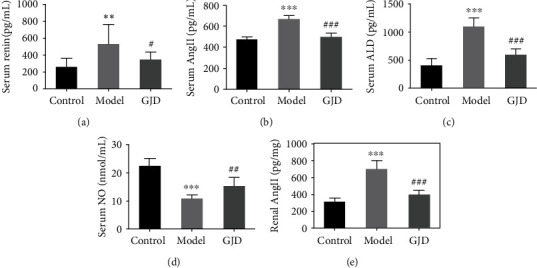
Effects of GJD on renin, Ang II, ALD, and NO expression in serum and Ang II in kidney tissue of high-salt induced hypertensive rats (*n* = 7). (a) Serum renin level. (b) Serum Ang II level. (c) Serum ALD level. (d) Serum NO level. (e) Kidney tissue Ang II level. ^∗^*P* < 0.05, ^∗∗^*P* < 0.01, and ^∗∗∗^*P* < 0.001 compared to the control group; ^#^*P* < 0.05, ^##^*P* < 0.01, and ^###^*P* < 0.001 compared to the model group.

The renin-angiotensin-aldosterone system plays an extremely important in the pathogenesis of primary hypertension, and NO is an important vasodilator factor in the body, and their expression changes in serum can reflect the development trend of primary hypertension. As shown in Figure 7, the expressions of renin, Ang II, and ALD in the model group's serum were increased significantly (*P* < 0.01, 0.001, and 0.001) in comparison to the control group, and the expression of NO was significantly decreased (*P* < 0.001). Compared with the model group, the expressions of renin, Ang II, and ALD in the serum of the GJD group were significantly decreased (*P* < 0.05, 0.001, and 0.001), and the expression of NO was significantly increased (*P* < 0.01). The expression of Ang II in kidney tissue of rats in the group was also significantly decreased (*P* < 0.001). The results indicate that GJD may reduce blood pressure by inhibiting the activation of the renin-angiotensin-aldosterone system and upregulating NO expression.

#### 3.4.3. Effect of GJD on the Expression of AGTR1 and eNOS

**Figure 8:**
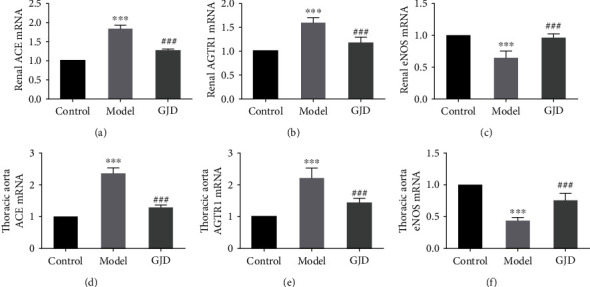
Effects of GJD on the expression of ACE (a, d), AGTR1 (b, e), and eNOS (c, f) mRNA in kidney and thoracic aorta of hypertensive rats (*n* = 7) induced by high-salt based on RT-qPCR. ∗*P* < 0.05, ∗∗*P* < 0.01, and ∗∗∗*P* < 0.001 compared with the control group; ^#^*P* < 0.05, ^##^*P* < 0.01, and ^###^*P* < 0.001 compared with the model group.

**Figure 9:**
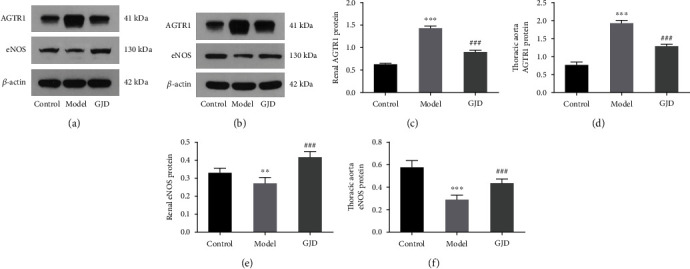
Effects of GJD on the expression of AGTR1 (a–d) and eNOS (a, b, e, and f) protein in the kidney (a) and thoracic aorta (b) of hypertensive rats induced by high-salt based on western blot analysis. ^∗^*P* < 0.05, ^∗∗^*P* < 0.01, and ^∗∗∗^*P* < 0.001 compared with the control group; ^#^*P* < 0.05, ^##^*P* < 0.01, and ^###^*P* < 0.001 compared with the model group.

After preliminary verification based on network pharmacology analysis and molecular docking, this study found that AGTR1 and eNOS play key roles in GJD's antihypertension. The results of RT-qPCR revealed that the expression of ACE and AGTR1 mRNA in the GJD group's thoracic aorta and kidney was inhibited. In contrast, the expression of eNOS mRNA was increased. Western blot investigations showed that the GJD group's expression of AGTR1 protein in the thoracic aorta and kidney was significantly lower than that of the model group, whereas eNOS expression was significantly higher. The result is shown in Figures 8 and 9.

## 4. Discussion

Network pharmacological analysis of Chinese medicines is becoming a research tool for studying the influence of Chinese medicines on diseases, intending to reveal complicated components and unexplored targets [44–46]. The GJD was developed to treat primary hypertension; however, the underlying mechanism remains unknown. The active components, targets, and signaling pathways of GJD in treating primary hypertension were predicted using network pharmacology, and the putative molecular mechanism was examined using molecular docking and animal experiments.

In this study, the network pharmacology results showed that 214 possible GJD targets for the treatment of hypertension were identified. Moreover, 35 hub targets were also uncovered in the PPI network diagram of potential targets, including STAT3, AKT1, APP, MAPK1, TP53, EP300, JUN, RELA, CXCL8, TNF, and EDN1. Based on the diagram of the GJD active ingredient-key target-pathway network, 11 components that were identified as active (quercetin, daidzein, ursolic acid, wogonin, luteolin, tanshinone IIA, puerarin, kaempferol, cryptotanshinone, beta carotene, and baicalein) are correlated with major targets of GJD in the treatment of hypertension, and it can be utilized as major components that play a pivotal role.

According to the KEGG analysis, the Toll-like receptor, MAPK, PI3K-Akt, TNF, NOD-like, NF-*κ*B, and renin-angiotensin signaling pathways are major targets of GJD in the treatment of hypertension. Ang II is the most powerful vasoconstrictor molecule in RAAS, and it causes hypertension through oxidative activation and inflammatory response. Ang II may also change myocardial and blood vessels through controlling ALD production [47]. In this regard, the abnormal activation of the RAAS system would lead to hypertension and a wide range of cardiovascular diseases.

A molecular docking verification was conducted on 35 key targets and 11 active ingredients. The results indicate the AGTR1, AKT1 with puerarin, EDNRA with tanshinone IIA, MAPK14 with daidzein, MAPK8 with ursolic acid, and CHRM2 with cryptotanshinone had a high binding activity to targets with active ingredients. The smaller the binding energy, the more stable the ligand-receptor binding conformation, and the greater the possibility of interaction [35]. In our study, the binding of AGTR1 to puerarin is mainly through five hydrogen bond interactions with ASP16, ARG23, TYR92, ASP1281, and hydrophobic binding ILE1288, and its binding energy reaches -9.2 kcal·mol^−1^, indicating the binding of AGTR1 to puerarin is very active. Therefore, we performed experimental validation of the AGTR1 target.

When it comes to hypertensive individuals, antihypertensive medication is likely to lower the occurrence and death from disorders such as cardiovascular and cerebrovascular. Many clinical studies have pointed out that lowering the systolic blood pressure (SBP) by 10 mmHg or the diastolic blood pressure (DBP) at least by 5 mmHg may reduce the risk of mortality by 10%-15%, resulting in a 35% of reduced risk of stroke, 20% of lower risk of coronary heart disease, and 40% of lower risk of heart failure [48]. There are many variables that impact the incidence of critical hypertension; for instance, the excessive intake of salt is the most prevalent cause of hypertension, especially among Chinese citizens [49]. Excessive salt intake raises blood pressure, increasing the risk of hypertension and many other cardiovascular complications [49]. In this study, the SBP and DBP were significantly reduced in the GJD group, indicating that GJD has a significant antihypertensive effect. In addition, the LVMI of the rats in the GJD group was also lower than that in the model group, so GJD may also improve left ventricular hypertrophy, a complication of hypertension.

Many studies have been carried out recently to examine the association between excessive salt intake and elevated blood pressure; how increased salt consumption causes hypertension is not yet exclusively understood. Several studies have confirmed that high-salt foods damage the heart, renal, and vascular tissue and change the function of the renin-angiotensin system mechanism, thereby changing the levels of Ang II, ALD, renin, and AGTR1 in high-salt rats [36, 50, 51]. AGTR1 is regarded as an angiotensin II type 1 receptor, and the major active substance of the renin-angiotensin-aldosterone system (RAAS) is Ang II. Ang II influences the development of vasoconstriction and aldosterone production in the adrenal cortex and causes hypertension via oxidative activation and inflammatory reactions [52]. A high-salt diet may influence the activation of the renal RAAS system and up the production of Ang II, leading to hypertension and renal damage in rats [53]. Puerarin is often used to treat various diseases, including hypertension, cardiac hypertrophy, atherosclerosis, diabetes-related cardiovascular complications, and stroke. Many experimental studies have indicated that puerarin can cause a reduction of AGTR1 expression in hypertension animal models. Puerarin can also boost cardiovascular function through increasing the eNOS expression and serum NO content via the PI3K/Akt pathway [20]. Vascular endothelial dysfunction can lead to various cardiovascular diseases, including hypertension, atherosclerosis, and thrombosis. Tanshinone IIA can decrease blood pressure and enhance endothelial-dependent vasodilation by quashing ET-1 expression induced by oxidative stress and increasing NO [19]. In addition, tanshinone IIA may inhibit the expression of EDNRA, upsurge the expression of EDNRB and eNOS, and guard against the endothelial function of the rat aorta [54]. GJD was observed to reduce the expression of AGTR1 mRNA and protein in the kidney and thoracic aorta of high-salt hypertension model rats while increasing the expression of eNOS mRNA and protein in the kidney and thoracic aorta.

The current study acknowledges that the GJD reduces the SBP, DBP, heart rate, and LVMI in high-salt hypertensive model rats and alters blood pressure-related biomarkers in rat serum. Renin, Ang II, and ALD expressions in the GJD group are also considerably reduced, but NO expression is highly increased. The results of RT-qPCR and WB indicate that GJD could lower the expression of AGTR1 mRNA and protein in the kidney and thoracic aorta of high-salt hypertension model rats, whereas it enhances the expression of eNOS mRNA and protein in the kidney and thoracic aorta, which concurs with the results of network pharmacology and a molecular docking prediction. Hence, this study confirms a few key targets and particular pathways.

## 5. Conclusion

As such, it can be concluded that the network pharmacology and molecular docking methods were utilized to investigate the intervention mechanism of GJD in primary hypertension. The research uncovered that the key active ingredients of GJD in managing primary hypertension are puerarin, tanshinone IIA, daidzein, ursolic acid, and cryptotanshinone, which are mainly expected to contain a regulatory function in conjunction with AGTR1, AKT1, EDNRA, MAPK14, MAPK8, and CHRM2. We identified these 5 key active ingredients in GJD by HPLC. Based on the results, the high-salt rat model experiment confirmed that GJD has an obvious antihypertensive effect by significantly reducing systolic and diastolic blood pressure, constrains the overactivation of the renin-angiotensin pathway, reduces the expression of AGTR1 in the kidney and thoracic aorta, and enhances the expression of eNOS. The current study discovered that GJD could be a potential therapeutic drug for primary hypertension, establishing a new strategy for the investigation and mechanism research of traditional Chinese medicine in treating hypertension.

## Figures and Tables

**Table 1:** Primer's list for RT-qPCR.

Gene	Sequences of primers
AGTR1	Forward: 5′-AATATTTGGAAACAGCTTGGT-3′
	Reverse: 5′-ATGATGATGCAGGTGACTTTG-3′
ACE	Forward: 5′-CCAACAAGACTGCCACCTG-3′
	Reverse: 5′-GTACTGGTGACATCGAGGTTG-3′
eNOS	Forward: 5′-GGATTCTGGCAAGACCGATTAC-3′
	Reverse: 5′-GGTGAGGACTTGTCCAAACACT-3′
GAPDH	Forward: 5′-TGCACCACCAACTGCTTAG-3′
	Reverse: 5′-GATGCAGGGATGATGTTC-3′

**Table 2:** PDB ID of the target proteins.

Target	PDB ID	Target	PDB ID	Target	PDB ID	Target	PDB ID
F2	8KME	EP300	6V8K	AGTR1	6OS2	JUN	5FV8
ESR1	7KCA	VEGFA	6V7K	FN1	6MSV	IL6	5FUC
TNF	7JRA	RHOA	6V6U	APP	6GFI	TGFB1	5FFO
CTNNB1	7AFW	MAPK1	6SLG	MYC	6G6K	STAT1	3WWT
EGFR	7A2A	TP53	6SL6	EDN1	6DK5	EGF	3NJP
MAPK14	6ZWP	AKT1	6S9W	ITGB3	6BXJ	CXCL10	1O7Y
MAPK8	6ZR5	STAT3	6QHD	CHRM2	5ZKC	RELA	1NFI
IL1B	6Y8M	CASP8	6PX9	IGF1	5U8Q	FOS	1A02
CXCL8	6WZM	CCND1	6P8E	EDNRA	5GLH		

## Data Availability

The data used to support the findings of this study are included within Supplementary Material (1) and Supplementary Material (2).

## Abbreviations

ADME: Absorption, distribution, metabolism, and excretion
AGTR1: Angiotensin II receptor type 1
DBP: Diastolic blood pressure
eNOS: Nitric oxide synthase 3
GJD: Gedan Jiangya Decoction
GO: Gene Ontology
HR: Heart rate
HPLC: High-performance liquid chromatography
KEGG: Kyoto Encyclopedia of Genes and Genomes
LVMI: Left ventricular mass index
SBP: Systolic blood pressure
STRING: Search Tool for the Retrieval of Interacting Genes/Proteins
TCM: Traditional Chinese medicine
TCMSP: Traditional Chinese Medicine Systems Pharmacology Database and Analysis Platform.

## Supplementary Materials

Supplementary Materials Supplementary Material (1): all the experiment data. Supplementary Material (2): network pharmacology analysis.
