# Supplementary material for: Network Pharmacology and Molecular Docking-Based Mechanism Study to Reveal Antihypertensive Effect of Gedan Jiangya Decoction
Source: Biomed Res Int. 2022 Aug 22;2022:3353464. doi: 10.1155/2022/3353464 (PMC9423997; doi:10.1155/2022/3353464)
Supplement: Supplementary Materials — Supplementary Material (1): all the experiment data. Supplementary Material (2): network pharmacology analysis. [file 3353464.f1.zip › Supplementary Material (1) (1).pdf]

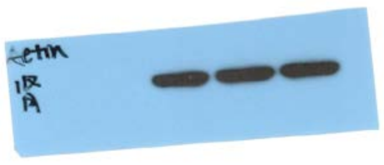

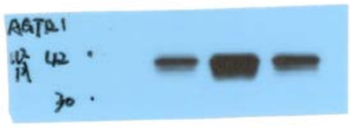

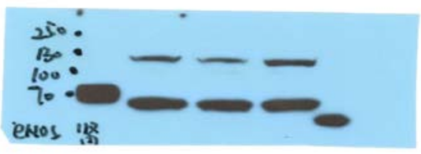

AGTR1	Control	Model	GJD
	0.62	1.42	0.90
eNOS	Control	Model	GJD
	0.33	0.27	0.42

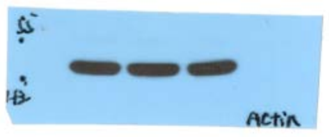

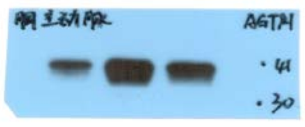

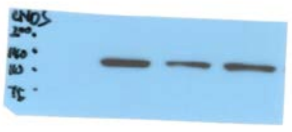

AGTR1	Control	Model	GJD
	0.76	1.92	1.29
eNOS	Control	Model	GJD
	0.57	0.29	0.44

kidney	AGTR1	Control	Model	GJD
	1	1	1.73	1.07
	2	1	1.59	1.37
	3	1	1.47	1.22
	4	1	1.69	1.04
	5	1	1.67	1.03
	6	1	1.48	1.21
	7	1	1.57	1.23
	Mean	1.00	1.60	1.17

kidney	ACE	Control	Model	GJD
	1	1	1.99	1.33
	2	1	1.83	1.26
	3	1	1.82	1.26
	4	1	1.9	1.25
	5	1	1.77	1.31
	6	1	1.86	1.22
	7	1	1.71	1.2
	Mean	1.00	1.84	1.26

kidney	eNOS	Control	Model	GJD
	1	1	0.59	1.01
	2	1	0.67	1.06
	3	1	0.45	0.99
	4	1	0.83	0.94
	5	1	0.66	0.93
	6	1	0.65	0.92
	7	1	0.64	0.9
	Mean	1.00	0.64	0.96

aorta	AGTR1	Control	Model	GJD
	1	1	2.33	1.33
	2	1	1.91	1.44
	3	1	1.96	1.31
	4	1	2.44	1.19
	5	1	2.07	1.53
	6	1	2.78	1.67
	7	1	1.97	1.49
	Mean	1.00	2.21	1.42

aorta	ACE	Control	Model	GJD
	1	1	2.5	1.35
	2	1	2.39	1.27
	3	1	2.44	1.21
	4	1	2.27	1.29
	5	1	2.57	1.3
	6	1	2.32	1.19
	7	1	2.04	1.4
	Mean	1.00	2.36	1.29

aorta	eNOS	Control	Model	GJD
	1	1	0.45	0.89
	2	1	0.46	0.86
	3	1	0.39	0.79
	4	1	0.53	0.81
	5	1	0.38	0.67
	6	1	0.42	0.66
	7	1	0.4	0.59
	Mean	1.00	0.43	0.75

pg/ml	Control	Model	GJD
1	289.38	951.90	324.35
2	132.85	741.92	499.81
3	205.41	522.16	389.07
4	429.76	428.16	268.70
5	266.74	307.29	278.14
6	150.14	393.17	414.54
7	331.55	370.66	256.92
Mean	257.97	530.75	347.36
SD	97.13	216.15	83.82

pg/ml	Control	Model	GJD
1	496.23	679.49	465.62
2	496.05	688.67	515.43
3	444.42	625.55	539.35
4	438.62	625.08	547.86
5	492.25	724.70	454.57
6	477.28	669.04	469.04
7	469.21	648.17	474.30
Mean	473.44	665.82	495.17
SD	22.27	33.34	35.41

pg/ml	Control	Model	GJD
1	510.26	1231.44	644.25
2	463.36	1098.03	498.16
3	360.43	1207.15	646.24
4	251.14	806.49	578.71
5	375.17	1227.39	413.86
6	583.78	981.36	701.78
7	334.27	1137.89	697.08
Mean	411.20	1098.54	597.15
SD	105.38	144.73	99.50

pg/mg	Control	Model	GJD
1	322.56	617.68	436.14
2	356.91	711.65	440.24
3	261.76	848.69	358.35
4	276.41	638.90	450.63
5	326.47	644.81	391.65
6	384.31	606.47	350.45
7	249.27	837.75	417.69
Mean	311.10	700.85	406.45
SD	46.73	95.26	37.36

µmol/L	Control	Model	GJD
1	21.38	12.38	13.48
2	24.03	11.19	19.68
3	21.68	9.50	12.49
4	21.35	12.53	13.65
5	18.17	11.26	12.01
6	24.20	9.65	17.00
7	26.47	9.04	18.45
Mean	22.47	10.79	15.25
SD	2.48	1.31	2.85

Baseline	Control	Model	GJD	2 weeks	Control	Model	GJD	4 weeks	Control	Model	GJD	6 weeks	Control	Model	GJD	8 weeks	Control	Model	GJD	10 weeks	Control	Model	GJD	12 weeks	Control	Model	GJD
1	123	114	144	1	126	132	143	1	129	153	162	1	134	165	173	1	140	165	180	1	135	174	163	1	131	171	135
2	123	142	126	2	128	156	151	2	144	161	156	2	147	174	170	2	126	177	182	2	138	174	158	2	119	162	133
3	113	122	131	3	114	147	140	3	125	154	152	3	119	172	172	3	144	181	173	3	129	169	160	3	117	171	139
4	114	138	137	4	115	142	155	4	120	149	160	4	118	176	168	4	114	183	183	4	128	168	173	4	129	173	123
5	119	140	119	5	131	157	133	5	133	166	148	5	132	171	171	5	139	183	167	5	129	174	164	5	144	171	131
6	139	126	136	6	137	148	159	6	135	142	155	6	140	151	162	6	128	171	171	6	113	170	175	6	124	170	142
7	131	136	138	7	127	151	142	7	125	160	161	7	120	173	166	7	116	176	181	7	121	163	170	7	126	165	145
Mean	123.19	131.14	133.14	Mean	125.43	147.57	146.14	Mean	130.14	155.00	156.29	Mean	130.09	168.86	168.76	Mean	129.62	176.68	176.86	Mean	127.53	170.46	166.16	Mean	127.21	168.98	135.31
SD	8.71	9.95	7.75	SD	7.69	7.98	8.49	SD	7.38	7.48	4.74	SD	10.62	7.95	3.64	SD	10.91	6.05	5.90	SD	7.94	3.89	6.21	SD	8.18	3.81	6.67

Baseline	Control	Model	GJD	2 weeks	Control	Model	GJD	4 weeks	Control	Model	GJD	6 weeks	Control	Model	GJD	8 weeks	Control	Model	GJD	10 weeks	Control	Model	GJD	12 weeks	Control	Model	GJD
1	92	85	105	1	91	100	111	1	94	105	120	1	98	122	121	1	100	132	127	1	95	126	127	1	94	114	102
2	89	101	94	2	95	106	104	2	102	110	105	2	111	123	125	2	89	116	126	2	101	117	122	2	86	120	97
3	81	91	91	3	83	98	113	3	88	109	121	3	79	125	132	3	106	126	130	3	90	126	118	3	83	123	97
4	81	99	96	4	80	109	99	4	84	112	108	4	86	129	128	4	84	124	128	4	97	118	128	4	99	117	84
5	97	113	85	5	99	107	91	5	101	114	102	5	98	117	126	5	101	130	123	5	92	134	123	5	96	119	92
6	103	84	96	6	100	113	106	6	98	104	109	6	93	111	121	6	93	125	120	6	88	118	121	6	95	116	105
7	94	98	99	7	94	118	105	7	91	127	117	7	87	137	122	7	78	127	123	7	84	119	116	7	95	117	107
Mean	91.05	95.67	95.19	Mean	91.71	107.29	104.14	Mean	94.00	111.57	111.71	Mean	92.98	123.56	125.04	Mean	92.92	125.62	125.28	Mean	92.47	122.53	122.27	Mean	92.43	118.08	97.69
SD	7.70	9.31	5.93	SD	7.09	6.45	6.85	SD	6.26	7.11	7.00	SD	9.41	7.56	3.86	SD	9.43	4.79	3.45	SD	5.38	5.81	4.05	SD	5.48	2.94	7.46

Baseline	Control	Model	GJD	2 weeks	Control	Model	GJD	4 weeks	Control	Model	GJD	6 weeks	Control	Model	GJD	8 weeks	Control	Model	GJD	10 weeks	Control	Model	GJD	12 weeks	Control	Model	GJD
1	398	316	411	1	386	369	423	1	388	387	426	1	370	396	435	1	392	434	417	1	415	425	389	1	372	410	364
2	395	421	413	2	415	446	405	2	410	448	400	2	409	474	403	2	377	449	415	2	406	420	419	2	407	422	406
3	369	407	377	3	399	412	367	3	399	410	372	3	394	406	404	3	405	410	401	3	414	394	412	3	390	427	381
4	414	429	383	4	410	411	386	4	402	398	377	4	412	377	382	4	395	438	377	4	408	402	395	4	416	447	385
5	404	377	383	5	400	389	416	5	397	411	417	5	389	420	408	5	366	406	403	5	336	415	405	5	382	414	364
6	414	453	416	6	388	410	429	6	376	396	424	6	398	384	420	6	409	437	430	6	381	420	407	6	380	430	395
7	384	426	419	7	371	427	395	7	381	425	401	7	375	462	381	7	407	453	459	7	379	453	404	7	386	378	385
Mean	396.90	404.14	400.29	Mean	395.57	409.14	403.00	Mean	393.29	410.71	402.43	Mean	392.53	416.95	404.95	Mean	393.17	432.56	414.47	Mean	391.23	418.30	404.29	Mean	390.48	418.12	382.91
SD	14.96	41.88	16.97	SD	13.99	23.01	20.35	SD	11.21	19.03	20.05	SD	14.67	34.87	18.00	SD	15.00	16.64	23.57	SD	26.36	17.32	9.32	SD	14.23	19.94	14.02

Baseline	Control	Model	GJD	2 weeks	Control	Model	GJD	4 weeks	Control	Model	GJD	6 weeks	Control	Model	GJD	8 weeks	Control	Model	GJD	10 weeks	Control	Model	GJD	12 weeks	Control	Model	GJD
1	199.8	185.3	189.2	1	253.4	257.7	266.6	1	302.6	298.8	313.3	1	361.6	348.1	377.7	1	393.7	401.2	409.5	1	417.4	415.8	445.7	1	425.3	428.8	469.2
2	187.6	195.1	190.5	2	260.6	265.6	255.7	2	290.9	307.8	297.5	2	329.7	341.7	307.5	2	372.3	393.8	370.8	2	400.2	417.3	391.3	2	402.4	444.4	410.5
3	200.6	182.7	189.4	3	260.5	258.7	252.5	3	300.1	318.1	281.4	3	386.5	307.3	329.9	3	412.4	387.9	399.9	3	438.9	427.5	423.6	3	449.3	452.1	438.2
4	173.6	187.5	185.5	4	246.6	266.8	244.4	4	305.7	319.1	276.7	4	377.5	326.4	335.4	4	416.9	365.7	406.4	4	435.8	409.5	433.4	4	417.4	430.2	461.3
5	175.5	203.3	173.4	5	242.7	272.6	236.6	5	281.3	295.3	276.5	5	318.3	289.9	297.1	5	397.6	327.7	335.8	5	429.1	371.4	360.6	5	468.5	399.1	389.6
6	199.3	194.7	205.7	6	248.5	267.3	298.6	6	291.1	310.9	352.6	6	370.7	388.8	382.5	6	407.5	448.9	414.4	6	430.3	468	463.5	6	441.2	489.4	476.4
7	182.4	200.5	209.9	7	258.4	279.1	277.1	7	269.9	333.7	310.1	7	340.2	346.4	371.6	7	384.5	407.1	401.1	7	424.4	450.2	407.6	7	426.7	477.7	401.2
Mean	188.40	192.73	191.94	Mean	252.96	266.83	261.64	Mean	291.66	311.96	301.16	Mean	354.93	335.51	343.10	Mean	397.84	390.33	391.13	Mean	425.16	422.81	417.96	Mean	432.97	445.96	435.20
SD	10.82	7.22	11.42	SD	6.66	6.92	19.54	SD	11.77	12.13	25.31	SD	23.89	29.55	32.02	SD	14.72	34.59	26.09	SD	12.12	28.57	32.12	SD	20.26	28.47	32.49

Control	Weight/g	LV/mg	LVMI	Model	Weight/g	LV/mg	LVMI	GJD	Weight/g	LV/mg	LVMI
1	425.3	683.2	1.61	1	428.8	815	1.90	1	469.2	812.3	1.73
2	402.4	618.9	1.54	2	444.4	821.6	1.85	2	410.5	656.9	1.60
3	449.3	641	1.43	3	452.1	819.1	1.81	3	438.2	687.8	1.57
4	417.4	621	1.49	4	430.2	755.9	1.76	4	461.3	700.5	1.52
5	468.5	734	1.57	5	399.1	720.5	1.81	5	389.6	674.8	1.73
6	441.2	718.5	1.63	6	489.4	861.8	1.76	6	476.4	783.3	1.64
7	426.7	676	1.58	7	477.7	837.6	1.75	7	401.2	747.2	1.86
Mean	432.97	670.37	1.55	Mean	445.96	804.50	1.81	Mean	435.20	723.26	1.67
SD	20.26	42.28	0.07	SD	28.47	45.38	0.05	SD	32.49	54.29	0.11

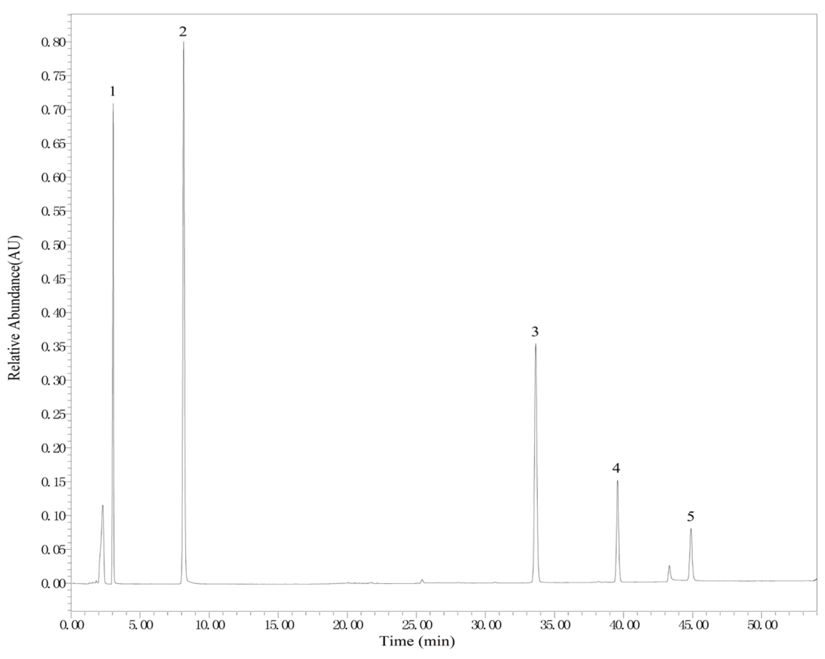

Standard chromatogram

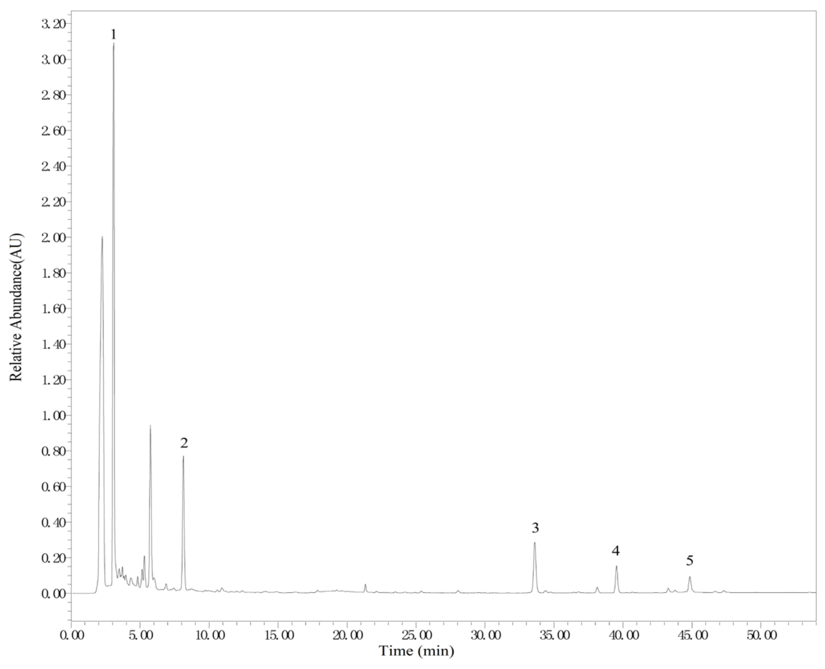

Sample chromatogram

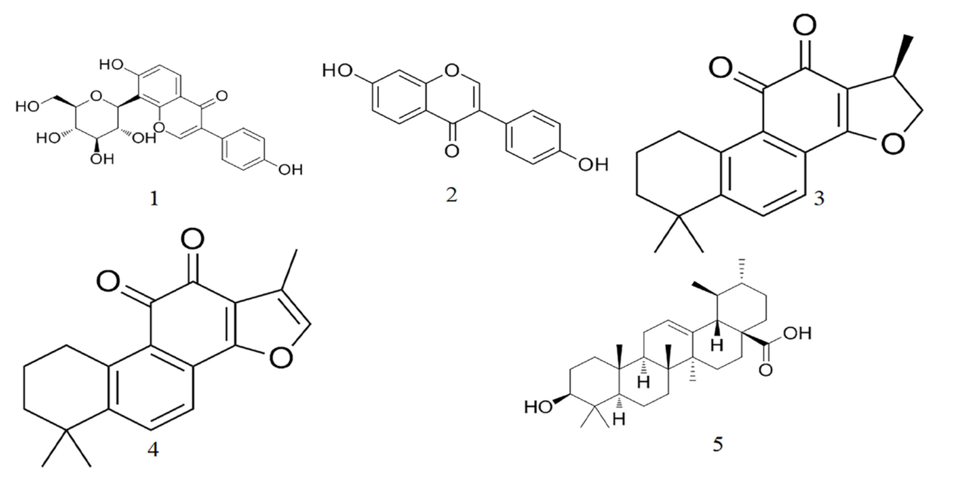

Ingredients

1. puerarin; 2. daidzein; 3. cryptotanshinone; 4. tanshinone IIA; 5. ursolic acid.
