# Supplementary material for: Network Pharmacology and Molecular Docking-Based Mechanism Study to Reveal Antihypertensive Effect of Gedan Jiangya Decoction
Source: Biomed Res Int. 2022 Aug 22;2022:3353464. doi: 10.1155/2022/3353464 (PMC9423997; doi:10.1155/2022/3353464)
Supplement: Supplementary Materials — Supplementary Material (1): all the experiment data. Supplementary Material (2): network pharmacology analysis. [file 3353464.f1.zip › Supplementary Material (2).pdf]

Mol	Symbol
cryptotanshinone	STAT3
cryptotanshinone	APP
cryptotanshinone	CCND1
cryptotanshinone	CHRM2
cryptotanshinone	EDN1
cryptotanshinone	RELA
cryptotanshinone	TNF
salvianolic acid a	AKT1
salvianolic acid a	CCND1
tanshinone iia	CHRM2
tanshinone iia	EDN1
tanshinone iia	EDNRA
tanshinone iia	F2
tanshinone iia	FOS
tanshinone iia	ITGB3
tanshinone iia	JUN
tanshinone iia	MYC
tanshinone iia	RELA
tanshinone iia	TP53
daidzein	EP300
daidzein	ESR1
daidzein	FOS
daidzein	IGF1
daidzein	IL6
daidzein	JUN
daidzein	MAPK14
daidzein	RELA
daidzein	RHOA
daidzein	STAT1
daidzein	TNF
daidzein	TP53
daidzein	VEGFA
wogonin	AKT1
wogonin	CCND1
wogonin	CXCL8
wogonin	ESR1
wogonin	FN1
wogonin	IL6
wogonin	JUN
wogonin	MAPK14
wogonin	RELA
wogonin	TNF
wogonin	TP53
puerarin	AGTR1
puerarin	AKT1
puerarin	CASP8
puerarin	ESR1
puerarin	FOS
puerarin	JUN
puerarin	RELA
puerarin	STAT3
puerarin	TNF
puerarin	VEGFA
epiquinidine	CHRM2
epiquinidine	EGFR
ursolic acid	STAT3
ursolic acid	CASP8
ursolic acid	CCND1

ursolic acid	FOS
ursolic acid	IL1B
ursolic acid	IL6
ursolic acid	JUN
ursolic acid	MAPK8
ursolic acid	RELA
ursolic acid	TNF
ursolic acid	TP53
ursolic acid	VEGFA
beta-carotene	AKT1
beta-carotene	CASP8
beta-carotene	CTNNB1
beta-carotene	JUN
beta-carotene	MYC
beta-carotene	VEGFA
ICCB1_000076	MAPK14
ICCB1_000076	F2
ICCB1_000076	ESR1
Tetrahydroalstonine	F2
pteropodine	F2
pteropodine	ESR1
hirsutine	CHRM2
baicalein	AKT1
baicalein	FOS
baicalein	RELA
baicalein	TP53
baicalein	VEGFA
formononetin	JUN
formononetin	MAPK14
formononetin	F2
formononetin	ESR1
morin	EDN1
CHEMBL2430311	MAPK14
CHEMBL2430311	F2
CHEMBL2430311	ESR1
Syringetin	MAPK14
Syringetin	ESR1
3'-Methoxydaidzein	MAPK14
3'-Methoxydaidzein	ESR1
hirsutin_qt	MAPK14
dehydrodiconiferyl alcohol 4,γ'-di-	F2
dehydrodiconiferyl alcohol 4,γ'-di-	ESR1
(+)-Dehydrodiconiferyl alcohol	F2
(+)-Dehydrodiconiferyl alcohol	ESR1
geissoschizinc acid	F2
Rhynchophylline A	F2
Rhynchophylline A	ESR1
methyl (E)-2-[(2S,3Z,12bS)-3-ethyl	F2
methyl (E)-2-[(2S,3Z,12bS)-3-ethyl	ESR1
isopteropodine	F2
isopteropodine	ESR1
hirsutasideA	F2
Mitraphyllic acid	F2
Mitraphyllic acid	ESR1
corynantheine	F2
(1'R,3S,4a'S,5a'S,10a'R)-1'-methyl-:	F2
(1'R,3S,4a'S,5a'S,10a'R)-1'-methyl-:	ESR1
(2S,12bR)-methyl 2-((E)-1-oxobut-	F2
vincoside lactam_qt	F2

vincoside lactam_qt	ESR1
delta(sup 18)-Hirsutine	F2
isocorynantheic acid	F2
Dehydrotanshinone II A	F2
Dehydrotanshinone II A	ESR1
3α-hydroxytanshinone II a	F2
formyltanshinone	F2
Methylenetanshinquinone	F2
Methylenetanshinquinone	CHRM2
Przewaquinone B	F2
przewaquinone c	F2
przewaquinone c	CHRM2
(6S,7R)-6,7-dihydroxy-1,6-dimethy	F2
przewaquinone f	F2
tanshinaldehyde	F2
dan-shexinkum d	F2
dan-shexinkum d	ESR1
danshenspiroketallactone	CHRM2
danshenspiroketallactone	ESR1
danshenspiroketallactone	F2
dihydrotanshinlactone	F2
dihydrotanshinlactone	ESR1
ferruginol	F2
ferruginol	CHRM2
Isotanshinone II	F2
Isotanshinone II	ESR1
Isotanshinone II	CHRM2
miltionone II	F2
nortanshinone	F2
prolithospermic acid	F2
prolithospermic acid	ESR1
ZINC13341234	F2
ZINC13341234	ESR1
tanshindiol A	F2
salviolone	F2
salviolone	CHRM2
Tanshindiol B	F2
Przewaquinone E	F2
tanshinone IIB	F2
2-isopropyl-8-methylphenanthren	ESR1
2-isopropyl-8-methylphenanthren	CHRM2
4-methylenemiltirone	ESR1
4-methylenemiltirone	CHRM2
Salvilenone	ESR1
deoxyneocryptotanshinone	ESR1
deoxyneocryptotanshinone	CHRM2
epidanshenspiroketallactone	ESR1
epidanshenspiroketallactone	CHRM2
isocryptotanshinone	ESR1
isocryptotanshinone	CHRM2
miltionone I	ESR1
miltionone I	CHRM2
miltipolone	ESR1
Miltirone	ESR1
Miltirone	CHRM2
neocryptotanshinone ii	ESR1
neocryptotanshinone ii	CHRM2
tanshinone VI	ESR1
berberine	ESR1

coptisine	ESR1
epiberberine	ESR1
palmatine	ESR1
Inophyllum E	ESR1
Erythraline	CHRM2
(-)-Tabernemontanine	CHRM2
yohimbine	CHRM2
1,2,5,6-tetrahydrotanshinone	CHRM2
sugiol	CHRM2
quercetin	AKT1
quercetin	CASP8
quercetin	CCND1
quercetin	CXCL10
quercetin	CXCL8
quercetin	EGF
quercetin	EGFR
quercetin	F2
quercetin	FOS
quercetin	IL1B
quercetin	IL6
quercetin	JUN
quercetin	MAPK1
quercetin	MYC
quercetin	RELA
quercetin	STAT1
quercetin	TGFB1
quercetin	TNF
quercetin	TP53
quercetin	VEGFA
beta-sitosterol	CASP8
beta-sitosterol	CHRM2
beta-sitosterol	JUN
beta-sitosterol	TGFB1
kaempferol	AKT1
kaempferol	CHRM2
kaempferol	F2
kaempferol	JUN
kaempferol	MAPK8
kaempferol	RELA
kaempferol	STAT1
kaempferol	TNF
luteolin	AKT1
luteolin	APP
luteolin	CCND1
luteolin	EGFR
luteolin	IL6
luteolin	JUN
luteolin	MAPK1
luteolin	RELA
luteolin	TNF
luteolin	TP53
luteolin	VEGFA
3-beta-Hydroxymethyllenetanshiq	F2
ent-Epicatechin	ESR1
Stigmasterol	CHRM2
Pathways in cancer	AGTR1
Pathways in cancer	AKT1
Pathways in cancer	RHOA
Pathways in cancer	CCND1

Pathways in cancer	CASP8
Pathways in cancer	CTNNB1
Pathways in cancer	EDN1
Pathways in cancer	EDNRA
Pathways in cancer	EGF
Pathways in cancer	EGFR
Pathways in cancer	EP300
Pathways in cancer	ESR1
Pathways in cancer	F2
Pathways in cancer	FN1
Pathways in cancer	FOS
Pathways in cancer	IGF1
Pathways in cancer	IL6
Pathways in cancer	CXCL8
Pathways in cancer	JUN
Pathways in cancer	MYC
Pathways in cancer	MAPK1
Pathways in cancer	MAPK8
Pathways in cancer	RELA
Pathways in cancer	STAT1
Pathways in cancer	STAT3
Pathways in cancer	TGFB1
Pathways in cancer	TP53
Pathways in cancer	VEGFA
AGE-RAGE signaling pathway in di	AGTR1
AGE-RAGE signaling pathway in di	AKT1
AGE-RAGE signaling pathway in di	CCND1
AGE-RAGE signaling pathway in di	MAPK14
AGE-RAGE signaling pathway in di	EDN1
AGE-RAGE signaling pathway in di	FN1
AGE-RAGE signaling pathway in di	IL1B
AGE-RAGE signaling pathway in di	IL6
AGE-RAGE signaling pathway in di	CXCL8
AGE-RAGE signaling pathway in di	JUN
AGE-RAGE signaling pathway in di	MAPK1
AGE-RAGE signaling pathway in di	MAPK8
AGE-RAGE signaling pathway in di	RELA
AGE-RAGE signaling pathway in di	STAT1
AGE-RAGE signaling pathway in di	STAT3
AGE-RAGE signaling pathway in di	TGFB1
AGE-RAGE signaling pathway in di	TNF
AGE-RAGE signaling pathway in di	VEGFA
Hepatitis B	AKT1
Hepatitis B	CCND1
Hepatitis B	CASP8
Hepatitis B	MAPK14
Hepatitis B	EP300
Hepatitis B	FOS
Hepatitis B	IL6
Hepatitis B	CXCL8
Hepatitis B	JUN
Hepatitis B	MYC
Hepatitis B	MAPK1
Hepatitis B	MAPK8
Hepatitis B	RELA
Hepatitis B	STAT1
Hepatitis B	STAT3
Hepatitis B	TGFB1
Hepatitis B	TNF

Hepatitis B	TP53
kaposi sarcoma-associated herpes\	AKT1
kaposi sarcoma-associated herpes\	CCND1
kaposi sarcoma-associated herpes\	CASP8
kaposi sarcoma-associated herpes\	MAPK14
kaposi sarcoma-associated herpes\	CTNNB1
kaposi sarcoma-associated herpes\	EP300
kaposi sarcoma-associated herpes\	FOS
kaposi sarcoma-associated herpes\	IL6
kaposi sarcoma-associated herpes\	CXCL8
kaposi sarcoma-associated herpes\	JUN
kaposi sarcoma-associated herpes\	MYC
kaposi sarcoma-associated herpes\	MAPK1
kaposi sarcoma-associated herpes\	MAPK8
kaposi sarcoma-associated herpes\	RELA
kaposi sarcoma-associated herpes\	STAT1
kaposi sarcoma-associated herpes\	STAT3
kaposi sarcoma-associated herpes\	TP53
kaposi sarcoma-associated herpes\	VEGFA
Proteoglycans in cancer	AKT1
Proteoglycans in cancer	RHOA
Proteoglycans in cancer	CCND1
Proteoglycans in cancer	MAPK14
Proteoglycans in cancer	CTNNB1
Proteoglycans in cancer	EGFR
Proteoglycans in cancer	ESR1
Proteoglycans in cancer	FN1
Proteoglycans in cancer	IGF1
Proteoglycans in cancer	IL6
Proteoglycans in cancer	ITGB3
Proteoglycans in cancer	MYC
Proteoglycans in cancer	MAPK1
Proteoglycans in cancer	STAT3
Proteoglycans in cancer	TGFB1
Proteoglycans in cancer	TNF
Proteoglycans in cancer	TP53
Proteoglycans in cancer	VEGFA
Hepatitis C	AKT1
Hepatitis C	CCND1
Hepatitis C	CASP8
Hepatitis C	MAPK14
Hepatitis C	CTNNB1
Hepatitis C	EGF
Hepatitis C	EGFR
Hepatitis C	CXCL8
Hepatitis C	CXCL10
Hepatitis C	MYC
Hepatitis C	MAPK1
Hepatitis C	MAPK8
Hepatitis C	RELA
Hepatitis C	STAT1
Hepatitis C	STAT3
Hepatitis C	TNF
Hepatitis C	TP53
human cytomegalovirus infection	AKT1
human cytomegalovirus infection	RHOA
human cytomegalovirus infection	CCND1
human cytomegalovirus infection	CASP8
human cytomegalovirus infection	MAPK14

human cytomegalovirus infection	CTNNB1
human cytomegalovirus infection	EGFR
human cytomegalovirus infection	IL1B
human cytomegalovirus infection	IL6
human cytomegalovirus infection	CXCL8
human cytomegalovirus infection	ITGB3
human cytomegalovirus infection	MYC
human cytomegalovirus infection	MAPK1
human cytomegalovirus infection	RELA
human cytomegalovirus infection	STAT3
human cytomegalovirus infection	TNF
human cytomegalovirus infection	TP53
human cytomegalovirus infection	VEGFA
Toll-like receptor signaling pathwa	AKT1
Toll-like receptor signaling pathwa	CASP8
Toll-like receptor signaling pathwa	MAPK14
Toll-like receptor signaling pathwa	FOS
Toll-like receptor signaling pathwa	IL1B
Toll-like receptor signaling pathwa	IL6
Toll-like receptor signaling pathwa	CXCL8
Toll-like receptor signaling pathwa	CXCL10
Toll-like receptor signaling pathwa	JUN
Toll-like receptor signaling pathwa	MAPK1
Toll-like receptor signaling pathwa	MAPK8
Toll-like receptor signaling pathwa	RELA
Toll-like receptor signaling pathwa	STAT1
Toll-like receptor signaling pathwa	TNF
MAPK signaling pathway	AKT1
MAPK signaling pathway	MAPK14
MAPK signaling pathway	EGF
MAPK signaling pathway	EGFR
MAPK signaling pathway	FOS
MAPK signaling pathway	IGF1
MAPK signaling pathway	IL1B
MAPK signaling pathway	IL6
MAPK signaling pathway	JUN
MAPK signaling pathway	MYC
MAPK signaling pathway	MAPK1
MAPK signaling pathway	MAPK8
MAPK signaling pathway	RELA
MAPK signaling pathway	TGFB1
MAPK signaling pathway	TNF
MAPK signaling pathway	TP53
MAPK signaling pathway	VEGFA
Colorectal cancer	AKT1
Colorectal cancer	RHOA
Colorectal cancer	CCND1
Colorectal cancer	CTNNB1
Colorectal cancer	EGF
Colorectal cancer	EGFR
Colorectal cancer	FOS
Colorectal cancer	JUN
Colorectal cancer	MYC
Colorectal cancer	MAPK1
Colorectal cancer	MAPK8
Colorectal cancer	TGFB1
Colorectal cancer	TP53
Pancreatic cancer	AKT1
Pancreatic cancer	CCND1

Pancreatic cancer	EGF
Pancreatic cancer	EGFR
Pancreatic cancer	MAPK1
Pancreatic cancer	MAPK8
Pancreatic cancer	RELA
Pancreatic cancer	STAT1
Pancreatic cancer	STAT3
Pancreatic cancer	TGFB1
Pancreatic cancer	TP53
Pancreatic cancer	VEGFA
Fluid shear stress and atheroscleros	AKT1
Fluid shear stress and atheroscleros	RHOA
Fluid shear stress and atheroscleros	MAPK14
Fluid shear stress and atheroscleros	CTNNB1
Fluid shear stress and atheroscleros	EDN1
Fluid shear stress and atheroscleros	FOS
Fluid shear stress and atheroscleros	IL1B
Fluid shear stress and atheroscleros	ITGB3
Fluid shear stress and atheroscleros	JUN
Fluid shear stress and atheroscleros	MAPK8
Fluid shear stress and atheroscleros	RELA
Fluid shear stress and atheroscleros	TNF
Fluid shear stress and atheroscleros	TP53
Fluid shear stress and atheroscleros	VEGFA
Chagas disease	AKT1
Chagas disease	CASP8
Chagas disease	MAPK14
Chagas disease	FOS
Chagas disease	IL1B
Chagas disease	IL6
Chagas disease	CXCL8
Chagas disease	JUN
Chagas disease	MAPK1
Chagas disease	MAPK8
Chagas disease	RELA
Chagas disease	TGFB1
Chagas disease	TNF
TNF signaling pathway	AKT1
TNF signaling pathway	CASP8
TNF signaling pathway	MAPK14
TNF signaling pathway	EDN1
TNF signaling pathway	FOS
TNF signaling pathway	IL1B
TNF signaling pathway	IL6
TNF signaling pathway	CXCL10
TNF signaling pathway	JUN
TNF signaling pathway	MAPK1
TNF signaling pathway	MAPK8
TNF signaling pathway	RELA
TNF signaling pathway	TNF
IL-17 signaling pathway	CASP8
IL-17 signaling pathway	MAPK14
IL-17 signaling pathway	FOS
IL-17 signaling pathway	IL1B
IL-17 signaling pathway	IL6
IL-17 signaling pathway	CXCL8
IL-17 signaling pathway	CXCL10
IL-17 signaling pathway	JUN
IL-17 signaling pathway	MAPK1

IL-17 signaling pathway	MAPK8
IL-17 signaling pathway	RELA
IL-17 signaling pathway	TNF
Focal adhesion	AKT1
Focal adhesion	RHOA
Focal adhesion	CCND1
Focal adhesion	CTNNB1
Focal adhesion	EGF
Focal adhesion	EGFR
Focal adhesion	FN1
Focal adhesion	IGF1
Focal adhesion	IL6
Focal adhesion	ITGB3
Focal adhesion	JUN
Focal adhesion	MAPK1
Focal adhesion	MAPK8
Focal adhesion	VEGFA
c-type lectin receptor signaling pat	AKT1
c-type lectin receptor signaling pat	RHOA
c-type lectin receptor signaling pat	CASP8
c-type lectin receptor signaling pat	MAPK14
c-type lectin receptor signaling pat	IL1B
c-type lectin receptor signaling pat	IL6
c-type lectin receptor signaling pat	JUN
c-type lectin receptor signaling pat	MAPK1
c-type lectin receptor signaling pat	MAPK8
c-type lectin receptor signaling pat	RELA
c-type lectin receptor signaling pat	STAT1
c-type lectin receptor signaling pat	TNF
Epstein-Barr virus infection	AKT1
Epstein-Barr virus infection	CCND1
Epstein-Barr virus infection	CASP8
Epstein-Barr virus infection	MAPK14
Epstein-Barr virus infection	EP300
Epstein-Barr virus infection	IL6
Epstein-Barr virus infection	CXCL10
Epstein-Barr virus infection	JUN
Epstein-Barr virus infection	MYC
Epstein-Barr virus infection	MAPK8
Epstein-Barr virus infection	RELA
Epstein-Barr virus infection	STAT1
Epstein-Barr virus infection	STAT3
Epstein-Barr virus infection	TNF
Epstein-Barr virus infection	TP53
Pertussis	RHOA
Pertussis	MAPK14
Pertussis	FOS
Pertussis	IL1B
Pertussis	IL6
Pertussis	CXCL8
Pertussis	JUN
Pertussis	MAPK1
Pertussis	MAPK8
Pertussis	RELA
Pertussis	TNF
Influenza A	AKT1
Influenza A	MAPK14
Influenza A	EP300
Influenza A	IL1B

Influenza A	IL6
Influenza A	CXCL8
Influenza A	CXCL10
Influenza A	JUN
Influenza A	MAPK1
Influenza A	MAPK8
Influenza A	RELA
Influenza A	STAT1
Influenza A	TNF
Tuberculosis	AKT1
Tuberculosis	RHOA
Tuberculosis	CASP8
Tuberculosis	MAPK14
Tuberculosis	EP300
Tuberculosis	IL1B
Tuberculosis	IL6
Tuberculosis	MAPK1
Tuberculosis	MAPK8
Tuberculosis	RELA
Tuberculosis	STAT1
Tuberculosis	TGFB1
Tuberculosis	TNF
Osteoclast differentiation	AKT1
Osteoclast differentiation	MAPK14
Osteoclast differentiation	FOS
Osteoclast differentiation	IL1B
Osteoclast differentiation	ITGB3
Osteoclast differentiation	JUN
Osteoclast differentiation	MAPK1
Osteoclast differentiation	MAPK8
Osteoclast differentiation	RELA
Osteoclast differentiation	STAT1
Osteoclast differentiation	TGFB1
Osteoclast differentiation	TNF
human papillomavirus infection	AKT1
human papillomavirus infection	CCND1
human papillomavirus infection	CASP8
human papillomavirus infection	CTNNB1
human papillomavirus infection	EGF
human papillomavirus infection	EGFR
human papillomavirus infection	EP300
human papillomavirus infection	FN1
human papillomavirus infection	ITGB3
human papillomavirus infection	MAPK1
human papillomavirus infection	RELA
human papillomavirus infection	STAT1
human papillomavirus infection	TNF
human papillomavirus infection	TP53
human papillomavirus infection	VEGFA
foxo signaling pathway	AKT1
foxo signaling pathway	CCND1
foxo signaling pathway	MAPK14
foxo signaling pathway	EGF
foxo signaling pathway	EGFR
foxo signaling pathway	EP300
foxo signaling pathway	IGF1
foxo signaling pathway	IL6
foxo signaling pathway	MAPK1
foxo signaling pathway	MAPK8

foxo signaling pathway	STAT3
foxo signaling pathway	TGFB1
Breast cancer	AKT1
Breast cancer	CCND1
Breast cancer	CTNNB1
Breast cancer	EGF
Breast cancer	EGFR
Breast cancer	ESR1
Breast cancer	FOS
Breast cancer	IGF1
Breast cancer	JUN
Breast cancer	MYC
Breast cancer	MAPK1
Breast cancer	TP53
Endocrine resistance	AKT1
Endocrine resistance	CCND1
Endocrine resistance	MAPK14
Endocrine resistance	EGFR
Endocrine resistance	ESR1
Endocrine resistance	FOS
Endocrine resistance	IGF1
Endocrine resistance	JUN
Endocrine resistance	MAPK1
Endocrine resistance	MAPK8
Endocrine resistance	TP53
Human T-cell leukemia virus 1 infe	AKT1
Human T-cell leukemia virus 1 infe	CCND1
Human T-cell leukemia virus 1 infe	CTNNB1
Human T-cell leukemia virus 1 infe	EP300
Human T-cell leukemia virus 1 infe	FOS
Human T-cell leukemia virus 1 infe	IL6
Human T-cell leukemia virus 1 infe	JUN
Human T-cell leukemia virus 1 infe	MYC
Human T-cell leukemia virus 1 infe	MAPK1
Human T-cell leukemia virus 1 infe	MAPK8
Human T-cell leukemia virus 1 infe	RELA
Human T-cell leukemia virus 1 infe	TGFB1
Human T-cell leukemia virus 1 infe	TNF
Human T-cell leukemia virus 1 infe	TP53
Measles	AKT1
Measles	CCND1
Measles	CASP8
Measles	FOS
Measles	IL1B
Measles	IL6
Measles	JUN
Measles	MAPK8
Measles	RELA
Measles	STAT1
Measles	STAT3
Measles	TP53
Th17 cell differentiation	MAPK14
Th17 cell differentiation	FOS
Th17 cell differentiation	IL1B
Th17 cell differentiation	IL6
Th17 cell differentiation	JUN
Th17 cell differentiation	MAPK1
Th17 cell differentiation	MAPK8
Th17 cell differentiation	RELA

Th17 cell differentiation	STAT1
Th17 cell differentiation	STAT3
Th17 cell differentiation	TGFB1
Prolactin signaling pathway	AKT1
Prolactin signaling pathway	CCND1
Prolactin signaling pathway	MAPK14
Prolactin signaling pathway	ESR1
Prolactin signaling pathway	FOS
Prolactin signaling pathway	MAPK1
Prolactin signaling pathway	MAPK8
Prolactin signaling pathway	RELA
Prolactin signaling pathway	STAT1
Prolactin signaling pathway	STAT3
cryptotanshinone	DanShen
salvianolic acid a	DanShen
tanshinone iia	DanShen
ICCB1_000076	DanShen
Dehydrotanshinone II A	DanShen
3α-hydroxytanshinone II a	DanShen
formyltanshinone	DanShen
Methylenetanshinquinone	DanShen
Przewaquinone B	DanShen
przewaquinone c	DanShen
(6S,7R)-6,7-dihydroxy-1,6-dimethy	DanShen
przewaquinone f	DanShen
tanshinaldehyde	DanShen
dan-shexinkum d	DanShen
danshenspiroketallactone	DanShen
dihydrotanshinlactone	DanShen
ferruginol	DanShen
Isotanshinone II	DanShen
miltionone II	DanShen
nortanshinone	DanShen
prolithospermic acid	DanShen
ZINC13341234	DanShen
salviolone	DanShen
tanshindiol A	DanShen
Tanshindiol B	DanShen
Przewaquinone E	DanShen
tanshinone IIB	DanShen
2-isopropyl-8-methylphenanthren	DanShen
4-methylenemiltirone	DanShen
Salvilenone	DanShen
deoxyneocryptotanshinone	DanShen
epidanshenspiroketallactone	DanShen
isocryptotanshinone	DanShen
miltionone I	DanShen
miltipolone	DanShen
Miltirone	DanShen
neocryptotanshinone ii	DanShen
tanshinone VI	DanShen
1,2,5,6-tetrahydrotanshinone	DanShen
sugiol	DanShen
luteolin	DanShen
3-beta-Hydroxymethyllenetanshiq	DanShen
epiquinidine	DuZhong
beta-carotene	DuZhong
CHEMBL2430311	DuZhong
Syringetin	DuZhong

hirsutin_qt	DuZhong
dehydrodiconiferyl alcohol 4,γ'-di-	DuZhong
(+)-Dehydrodiconiferyl alcohol	DuZhong
Erythraline	DuZhong
(-)-Tabernemontanine	DuZhong
beta-sitosterol	DuZhong
quercetin	DuZhong
kaempferol	DuZhong
3-beta-Hydroxymethyllenetanshiq	DuZhong
ent-Epicatechin	DuZhong
daidzein	GeGen
puerarin	GeGen
formononetin	GeGen
3'-Methoxydaidzein	GeGen
beta-sitosterol	GeGen
Tetrahydroalstonine	GouTeng
pteropodine	GouTeng
hirsutine	GouTeng
geissoschizinc acid	GouTeng
Rhynchophylline A	GouTeng
methyl (E)-2-[(2S,3Z,12bS)-3-ethyl	GouTeng
isopteropodine	GouTeng
hirsutasideA	GouTeng
Mitraphyllic acid	GouTeng
corynantheine	GouTeng
(1'R,3S,4a'S,5a'S,10a'R)-1'-methyl-:	GouTeng
(2S,12bR)-methyl 2-((E)-1-oxobut-	GouTeng
vincoside lactam_qt	GouTeng
delta(sup 18)-Hirsutine	GouTeng
isocorynantheic acid	GouTeng
yohimbine	GouTeng
beta-sitosterol	GouTeng
quercetin	GouTeng
kaempferol	GouTeng
ent-Epicatechin	GouTeng
wogonin	NiuXi
baicalein	NiuXi
berberine	NiuXi
coptisine	NiuXi
epiberberine	NiuXi
palmatine	NiuXi
Inophyllum E	NiuXi
beta-sitosterol	NiuXi
quercetin	NiuXi
kaempferol	NiuXi
Stigmasterol	NiuXi
ursolic acid	XiaKuCao
morin	XiaKuCao
beta-sitosterol	XiaKuCao
quercetin	XiaKuCao
kaempferol	XiaKuCao
luteolin	XiaKuCao
Stigmasterol	XiaKuCao

Term	Type
cryptotanshinone	DanShen
salvianolic acid a	DanShen
tanshinone iia	DanShen
ICCB1_000076	DanShen
Dehydrotanshinone II A	DanShen
3α-hydroxytanshinone II a	DanShen
formyltanshinone	DanShen
Methylenetanshinquinone	DanShen
Przewaquinone B	DanShen
przewaquinone c	DanShen
(6S,7R)-6,7-dihydroxy-1,6-dimethyl·	DanShen
przewaquinone f	DanShen
tanshinaldehyde	DanShen
dan-shexinkum d	DanShen
danshenspiroketallactone	DanShen
dihydrotanshinlactone	DanShen
ferruginol	DanShen
Isotanshinone II	DanShen
miltionone II	DanShen
nortanshinone	DanShen
prolithospermic acid	DanShen
ZINC13341234	DanShen
salviolone	DanShen
tanshindiol A	DanShen
Tanshindiol B	DanShen
Przewaquinone E	DanShen
tanshinone IIB	DanShen
2-isopropyl-8-methylphenanthrene·	DanShen
4-methylenemiltirone	DanShen
Salvilenone	DanShen
deoxyneocryptotanshinone	DanShen
epidanshenspiroketallactone	DanShen
isocryptotanshinone	DanShen
miltionone I	DanShen
miltipolone	DanShen
Miltirone	DanShen
neocryptotanshinone ii	DanShen
tanshinone VI	DanShen
1,2,5,6-tetrahydrotanshinone	DanShen
sugiol	DanShen
luteolin	DanShen
3-beta-Hydroxymethyllenetanshiqui	DanShen
epiquinidine	DuZhong
beta-carotene	DuZhong
CHEMBL2430311	DuZhong
Syringetin	DuZhong
hirsutin_qt	DuZhong
dehydrodiconiferyl alcohol 4,γ'-di-C	DuZhong
(+)-Dehydrodiconiferyl alcohol	DuZhong
Erythraline	DuZhong
(-)-Tabernemontanine	DuZhong
beta-sitosterol	DuZhong
quercetin	DuZhong
kaempferol	DuZhong
3-beta-Hydroxymethyllenetanshiqui	DuZhong
ent-Epicatechin	DuZhong
daidzein	GeGen
puerarin	GeGen

formononetin	GeGen
3'-Methoxydaidzein	GeGen
beta-sitosterol	GeGen
Tetrahydroalstonine	GouTeng
pteropodine	GouTeng
hirsutine	GouTeng
geissoschizinc acid	GouTeng
Rhynchophylline A	GouTeng
methyl (E)-2-[(2S,3Z,12bS)-3-ethylic	GouTeng
isopteropodine	GouTeng
hirsutasideA	GouTeng
Mitraphyllic acid	GouTeng
corynantheine	GouTeng
(1'R,3S,4a'S,5a'S,10a'R)-1'-methyl-2-	GouTeng
(2S,12bR)-methyl 2-((E)-1-oxobut-2	GouTeng
vincoside lactam_qt	GouTeng
delta(sup 18)-Hirsutine	GouTeng
isocorynantheic acid	GouTeng
yohimbine	GouTeng
beta-sitosterol	GouTeng
quercetin	GouTeng
kaempferol	GouTeng
ent-Epicatechin	GouTeng
wogonin	NiuXi
baicalein	NiuXi
berberine	NiuXi
coptisine	NiuXi
epiberberine	NiuXi
palmatine	NiuXi
Inophyllum E	NiuXi
beta-sitosterol	NiuXi
quercetin	NiuXi
kaempferol	NiuXi
Stigmasterol	NiuXi
ursolic acid	XiaKuCao
morin	XiaKuCao
beta-sitosterol	XiaKuCao
quercetin	XiaKuCao
kaempferol	XiaKuCao
luteolin	XiaKuCao
Stigmasterol	XiaKuCao
Pathways in cancer	Pathway
AGE-RAGE signaling pathway in diak	Pathway
Hepatitis B	Pathway
kaposi sarcoma-associated herpesvi	Pathway
Proteoglycans in cancer	Pathway
Hepatitis C	Pathway
human cytomegalovirus infection	Pathway
Toll-like receptor signaling pathway	Pathway
MAPK signaling pathway	Pathway
Colorectal cancer	Pathway
Pancreatic cancer	Pathway
Fluid shear stress and atherosclerosis	Pathway
Chagas disease	Pathway
TNF signaling pathway	Pathway
IL-17 signaling pathway	Pathway
Focal adhesion	Pathway
c-type lectin receptor signaling path	Pathway
Epstein-Barr virus infection	Pathway

Pertussis	Pathway
Influenza A	Pathway
Tuberculosis	Pathway
Osteoclast differentiation	Pathway
human papillomavirus infection	Pathway
foxo signaling pathway	Pathway
Breast cancer	Pathway
Endocrine resistance	Pathway
Human T-cell leukemia virus 1 infect	Pathway
Measles	Pathway
Th17 cell differentiation	Pathway
Prolactin signaling pathway	Pathway
STAT3	gene
AKT1	gene
APP	gene
MAPK1	gene
TP53	gene
EP300	gene
JUN	gene
RELA	gene
CXCL8	gene
TNF	gene
EDN1	gene
VEGFA	gene
EGFR	gene
MAPK8	gene
CTNNB1	gene
MAPK14	gene
IL6	gene
F2	gene
EGF	gene
ESR1	gene
FOS	gene
RHOA	gene
AGTR1	gene
IGF1	gene
MYC	gene
CHRM2	gene
CXCL10	gene
FN1	gene
STAT1	gene
CASP8	gene
CCND1	gene
EDNRA	gene
IL1B	gene
ITGB3	gene
TGFB1	gene
GouTeng	durg1
GeGen	durg2
DuZhong	durg3
XiaKuCao	durg4
NiuXi	durg5
DanShen	durg6

ABCC1
ACACA
ACE2
ACHE
ADH1C
ADRA1A
ADRA1B
ADRA1D
ADRA2A
ADRA2B
ADRA2C
ADRB1
ADRB2
AGTR1
AKT1
ALOX5AP
APOB
APP
AR
BACE1
BAX
BCL2
BCL2L1
BIRC5
CA2
CALCR
CASP1
CASP3
CASP8
CASP9
CAT
CAV1
CCL2
CCND1
CD36
CD40LG
CDKN1A
CDKN1B
CDKN2A
CHEK2
CHRM1
CHRM2
CHRM3
CHRM4
CHRM5
CHRNA2
CHUK
CLDN4
COL1A1
COL3A1
CPT1A
CREB1
CRP
CSF2
CTNNB1
CTSD
CXCL10
CXCL11
CXCL8

CYCS
CYP1A1
CYP1A2
CYP1B1
CYP21A2
CYP3A4
DPP4
DRD2
DRD3
DRD4
DRD5
DUOX2
ECE1
EDN1
EDNRA
EGF
EGFR
EP300
ERBB2
ESR1
ESR2
F10
F2
F2R
F3
F7
FAS
FASLG
FASN
FN1
FOS
GH1
GHR
GJA1
GPT
GSK3B
GSR
GSTM1
GSTM2
GSTP1
HIF1A
HMOX1
HSD3B1
HSD3B2
HSPB1
HTR1A
HTR1B
HTR2A
HTR2B
HTR2C
HTR3A
ICAM1
IFNG
IGF1
IGF1R
IGF2
IGFBP3
IL10
IL1A

IL1B
IL2
IL4
IL6
INPPL1
INSR
ITGB3
JUN
KCNH2
KCNMA1
KDR
LDLR
LEPR
LPL
MAOB
MAPK1
MAPK14
MAPK8
MDK
MDM2
MET
MGAM
MMP1
MMP10
MMP2
MMP3
MMP9
MPO
MT-ND6
MTTP
MYC
NCF1
NFE2L2
NGF
NOS2
NOS3
NOX5
NQO1
NR3C1
NR3C2
NUF2
OPRM1
PARP1
PCNA
PDE3A
PDGFA
PECAM1
PGR
PIK3CG
PIM1
PLAT
PLAU
PON1
POR
PPARA
PPARD
PPARG
PPP3CA
PRKCA

PRKCB
PRKCD
PTEN
PTGER3
PTGES
PTGS1
PTGS2
PTPN1
RAD51
RAF1
RASSF1
RELA
RHOA
RUNX2
SELE
SELP
SERPINE1
SIRT1
SLC2A4
SLC6A2
SLC6A3
SLC6A4
SLPI
SOD1
SPP1
STAT1
STAT3
TFF1
TGFB1
THBD
TIMP2
TNF
TP53
TYR
VCAM1
VEGFA
XDH

Target	Degree
STAT3	49
MAPK1	45
AKT1	45
APP	45
TP53	42
EP300	40
JUN	40
RELA	35
TNF	33
CXCL8	33
EDN1	32
VEGFA	31
EGFR	30
MAPK8	30
MAPK14	29
CTNNB1	29
IL6	28
F2	27
EGF	26
RHOA	25
ESR1	25
FOS	25
AGTR1	23
IGF1	22
MYC	22
CHRM2	21
STAT1	20
FN1	20
CXCL10	20
TGFB1	19
CASP8	19
CCND1	19
IL1B	19
EDNRA	19
ITGB3	19
IL2	18
IL4	18
ADRA2C	18
ADRA2B	18
ADRA2A	18
CREB1	17
PRKCD	17
APOB	17
NR3C1	17
OPRM1	17
HTR1A	17
PRKCA	16
CDKN1A	16
CHRM1	16
CASP3	16
PTPN1	16
CXCL11	16
DRD2	16
HIF1A	15
PRKCB	15
IGF2	15
CCL2	15
F2R	15

ADRA1B	15
HTR2C	15
HTR2A	15
PTGER3	15
DRD3	15
CHRM4	15
HTR1B	15
DRD4	15
MMP9	14
ERBB2	14
IGF1R	14
AR	14
ADRB2	14
CHRM5	14
ADRA1D	14
HTR2B	14
CHRM3	14
ADRA1A	14
IGFBP3	13
PPARA	13
RAF1	13
BCL2	13
NOS3	13
IFNG	13
IL10	13
MMP2	12
BCL2L1	11
CSF2	11
GSK3B	11
PPARG	11
SIRT1	11
IL1A	11
MDM2	11
SERPINE1	10
CHUK	10
CYCS	10
PTGS2	9
NOS2	9
SPP1	9
CAV1	9
MMP3	9
PTEN	9
ICAM1	9
NGF	8
MET	8
TFF1	8
LPL	8
MMP1	8
HMOX1	8
VCAM1	8
NFE2L2	8
CDKN2A	8
CD40LG	7
CD36	7
HSPB1	7
PGR	7
KDR	7
PLAU	7
BAX	7

CDKN1B	7
PCNA	7
CYP1A1	7
GHR	6
GH1	6
LDLR	6
CASP1	6
INSR	6
CASP9	6
INPPL1	6
CAT	6
CPT1A	5
PECAM1	5
FASLG	5
CTSD	5
FAS	5
MMP10	5
TIMP2	5
CYP3A4	5
HSD3B2	5
HSD3B1	5
CHEK2	4
ESR2	4
PIK3CG	4
NCF1	4
POR	4
COL1A1	4
F10	4
PARP1	4
CYP1A2	4
SLC2A4	3
BIRC5	3
MPO	3
SOD1	3
RAD51	3
COL3A1	3
SELE	3
PPP3CA	3
F3	3
NR3C2	3
CALCR	3
DRD5	3
ADRB1	3
GJA1	2
PIM1	2
LEPR	2
RUNX2	2
CRP	2
PDGFA	2
PLAT	2
RASSF1	2
THBD	2
ACE2	2
SLC6A4	2
PTGES	2
NQO1	2
SLPI	2
PTGS1	2
GSTM1	2

F7	2
CYP21A2	2
BACE1	1
ECE1	1
PPARD	1
CA2	1
GPT	1
MTTP	1
PON1	1
HTR3A	1
SLC6A3	1
MGAM	1
SELP	1
NUF2	1
GSR	1
CYP1B1	1
GSTP1	1
DPP4	1
MAOB	1
GSTM2	1
XDH	1
FASN	1
ACACA	1

pathway	gene radio	LogP	count
blood vess	0.6	-23.92	21
vasculature	0.6	-23.54	21
cardiovasc	0.6	-23.54	21
blood vess	0.571429	-23.13	20
positive re	0.542857	-23.04	19
positive re	0.542857	-22.7	19
angiogene	0.542857	-22.57	19
positive re	0.542857	-22.44	19
positive re	0.542857	-22.31	19
response t	0.457143	-21.61	16
regulation	0.485714	-21.22	17
response t	0.457143	-21.2	16
response t	0.542857	-20.73	19
cellular res	0.514286	-19.41	18
cellular res	0.371429	-18.11	13
positive re	0.4	-17.86	14
epithelial c	0.428571	-17.82	15
cellular res	0.457143	-17.79	16
response t	0.485714	-17.59	17
negative re	0.485714	-17.45	17
cellular res	0.342857	-17.14	12
response t	0.342857	-17.01	12
cellular res	0.342857	-16.83	12
positive re	0.457143	-16.5	16
positive re	0.285714	-16.33	10
positive re	0.371429	-16	13
positive re	0.428571	-15.76	15
positive re	0.342857	-15.56	12
leukocyte	0.4	-15.23	14
regulation	0.4	-15.12	14

pathway	gene radic	LogP	count
platelet alp	0.2	-10.74	7
platelet alp	0.171429	-9.65	6
vesicle lum	0.257143	-9.58	9
secretory g	0.228571	-8.31	8
cytoplasm	0.228571	-8.14	8
transcriptio	0.2	-6.72	7
RNA polym	0.142857	-5.75	5
nuclear tra	0.142857	-5.55	5
cell-cell ju	0.171429	-5.17	6
coated ves	0.142857	-4.5	5
endoplasm	0.142857	-4.44	5
membrane	0.142857	-4.31	5
membrane	0.142857	-4.31	5
adherens j	0.171429	-4.24	6
membrane	0.142857	-4.23	5
anchoring	0.171429	-4.17	6
postsynaps	0.171429	-4.04	6
clathrin-co	0.114286	-4.01	4
dendrite	0.171429	-4	6
lamellipod	0.114286	-4	4
dendritic t	0.171429	-3.99	6
spindle	0.142857	-3.99	5
protein-DI	0.114286	-3.9	4
focal adhe	0.142857	-3.79	5
cell-substr	0.142857	-3.78	5
cell-substr	0.142857	-3.76	5
plasma me	0.171429	-3.65	6
Golgi lume	0.085714	-3.56	3
clathrin-co	0.085714	-3.38	3
glutamater	0.114286	-3.25	4

pathway	gene radic	LogP	count
transcriptio	0.371429	-12.58	13
cytokine re	0.285714	-12.08	10
receptor re	0.342857	-11.93	12
receptor li	0.314286	-10.98	11
cytokine a	0.228571	-9.39	8
RNA polyr	0.2	-9.32	7
activating	0.171429	-9.25	6
kinase bin	0.314286	-9.02	11
core prom	0.142857	-8.79	5
core prom	0.142857	-8.79	5
phosphata	0.2	-8.44	7
protein kir	0.285714	-8.3	10
chromatin	0.257143	-7.7	9
growth fac	0.171429	-7.69	6
protein ph	0.171429	-7.56	6
growth fac	0.171429	-7.33	6
nitric-oxid	0.085714	-6.83	3
RNA polyr	0.114286	-6.43	4
integrin bi	0.142857	-6.06	5
DNA-bind	0.2	-5.97	7
RNA polyr	0.085714	-5.85	3
heparin bi	0.142857	-5.75	5
repressing	0.114286	-5.74	4
MAP kinas	0.085714	-5.7	3
ubiquitin-l	0.171429	-5.67	6
cell adhesi	0.2	-5.55	7
tumor nec	0.085714	-5.11	3
glycosamir	0.142857	-5.03	5
histone de	0.114286	-4.92	4
sulfur com	0.142857	-4.77	5

pathway	gene radic	LogP	count
Pathways i	0.8	-40.8	28
AGE-RAGE	0.514286	-35.24	18
Hepatitis E	0.514286	-30.39	18
kaposi sarc	0.514286	-29.64	18
Proteoglyc	0.514286	-28.7	18
Hepatitis C	0.485714	-28.51	17
human cyt	0.514286	-27.92	18
Toll-like re	0.4	-25.11	14
MAPK sigr	0.485714	-23.66	17
Colorectal	0.371429	-23.42	13
Pancreatic	0.342857	-23.29	12
Fluid shear	0.4	-23.12	14
Chagas dis	0.371429	-22.65	13
TNF signal	0.371429	-22.59	13
IL-17 signa	0.342857	-21.19	12
Focal adhe	0.4	-20.52	14
c-type lect	0.342857	-20.42	12
Epstein-Ba	0.428571	-20.33	15
Pertussis	0.314286	-19.98	11
Influenza A	0.371429	-19.83	13
Tuberculos	0.371429	-19.63	13
Osteoclast	0.342857	-19.37	12
human pa	0.428571	-19.26	15
foxo signa	0.342857	-19.08	12
Breast can	0.342857	-18.82	12
Endocrine	0.314286	-18.8	11
Human T-	0.4	-18.57	14
Measles	0.342857	-18.32	12
Th17 cell c	0.314286	-18.26	11
Prolactin s	0.285714	-18.09	10

ingredients	Count
quercetin	20
daidzein	13
ursolic acid	12
wogonin	11
luteolin	11
tanshinone iia	10
puerarin	10
kaempferol	8
cryptotanshinone	7
beta-carotene	6
baicalein	5
beta-sitosterol	4
formononetin	4
ICCB1_000076	3
CHEMBL2430311	3
danshenspiroketallactone	3
Isotanshinone II	3
salvianolic acid a	2
epiquinidine	2
pteropodine	2
Syringetin	2
3'-Methoxydaidzein	2
dehydrodiconiferyl alcohol 4,γ'-di-C	2
(+)-Dehydrodiconiferyl alcohol	2
Rhynchophylline A	2
methyl (E)-2-[(2S,3Z,12bS)-3-ethylic	2
isopteropodine	2
Mitraphyllic acid	2
(1'R,3S,4a'S,5a'S,10a'R)-1'-methyl-2-	2
vincoside lactam_qt	2
Dehydrotanshinone II A	2
Methylenetanshinquinone	2
przewaquinone c	2
dan-shexinkum d	2
dihydrotanshinlactone	2
ferruginol	2
prolithospermic acid	2
ZINC13341234	2
salviolone	2
2-isopropyl-8-methylphenanthrene	2
4-methylenemiltirone	2
deoxyneocryptotanshinone	2
epidanshenspiroketallactone	2
isocryptotanshinone	2
miltionone Ⅰ	2
Miltirone	2
neocryptotanshinone ii	2
Tetrahydroalstonine	1
hirsutine	1
morin	1
hirsutin_qt	1
geissoschizinc acid	1
hirsutasideA	1
corynantheine	1
(2S,12bR)-methyl 2-((E)-1-oxobut-2	1
delta(sup 18)-Hirsutine	1
isocorynantheic acid	1
3α-hydroxytanshinone II a	1

formyltanshinone	1
Przewaquinone B	1
(6S,7R)-6,7-dihydroxy-1,6-dimethyl·	1
przewaquinone f	1
tanshinaldehyde	1
miltionone II	1
nortanshinone	1
tanshindiol A	1
Tanshindiol B	1
Przewaquinone E	1
tanshinone IIB	1
3-beta-Hydroxymethyllenetanshiqui	1
Salvilenone	1
miltipolone	1
tanshinone VI	1
berberine	1
coptisine	1
epiberberine	1
palmatine	1
Inophyllum E	1
ent-Epicatechin	1
Erythraline	1
(-)-Tabernemontanine	1
Stigmasterol	1
yohimbine	1
1,2,5,6-tetrahydrotanshinone	1
sugiol	1

Key Ingredients	GJD	Key targets																			
Quercetin	Uncaria rhynchophylla, Eucommia ulmoides, Prunella vulgaris, Achyranthes bidentata	AKT1	CASP8	CCND1	CXCL10	CXCL8	EGF	EGFR	F2	FOS	IL1B	IL6	JUN	MAPK1	MYC	RELA	STAT1	TGFB1	TNF	TP53	VEGFA
Daidzein	Pueraria lobata	EP300	ESR1	FOS	IGF1	IL6	JUN	MAPK14	RELA	RHOA	STAT1	TNF	TP53	VEGFA							
Ursolic acid	Prunella vulgaris	STAT3	CASP8	CCND1	FOS	IL1B	IL6	JUN	MAPK8	RELA	TNF	TP53	VEGFA								
Wogonin	Achyranthes bidentata	AKT1	CCND1	CXCL8	ESR1	FN1	IL6	JUN	MAPK14	RELA	TNF	TP53									
Luteolin	Salvia miltiorrhiza, Prunella vulgaris	AKT1	APP	CCND1	EGFR	IL6	JUN	MAPK1	RELA	TNF	TP53	VEGFA									
Tanshinone Ⅱ A	Salvia miltiorrhiza	CHRM2	EDN1	EDNRA	F2	FOS	ITGB3	JUN	MYC	RELA	TP53										
Puerarin	Pueraria lobata	AGTR1	AKT1	CASP8	ESR1	FOS	JUN	RELA	STAT3	TNF	VEGFA										
Kaempferol	Uncaria rhynchophylla, Eucommia ulmoides, Prunella vulgaris, Achyranthes bidentata	AKT1	CHRM2	F2	JUN	MAPK8	RELA	STAT1	TNF												
Cryptotanshinone	Salvia miltiorrhiza	STAT3	APP	CCND1	CHRM2	EDN1	RELA	TNF													
Beta-carotene	Eucommia ulmoides	AKT1	CASP8	CTNNB1	JUN	MYC	VEGFA														
Baicalein	Achyranthes bidentata	AKT1	FOS	RELA	TP53	VEGFA															
